# Salient Object Detection Techniques in Computer Vision—A Survey

**DOI:** 10.3390/e22101174

**Published:** 2020-10-19

**Authors:** Ashish Kumar Gupta, Ayan Seal, Mukesh Prasad, Pritee Khanna

**Affiliations:** 1 PDPM-Indian Institute of Information Technology, Design and Manufacturing, Jabalpur, Dumna Airport Road, Jabalpur 482005, India; ashishkumargupta@iiitdmj.ac.in (A.K.G.); ayan@iiitdmj.ac.in (A.S.); 2 Centre for Artificial Intelligence, School of Computer Science, FEIT, University of Technology Sydney, Broadway, Sydney, NSW 2007, Australia; Mukesh.Prasad@uts.edu.au

**Keywords:** salient object detection, saliency cues, conventional salient object detection models, deep learning-based salient object detection models

## Abstract

Detection and localization of regions of images that attract immediate human visual attention is currently an intensive area of research in computer vision. The capability of automatic identification and segmentation of such salient image regions has immediate consequences for applications in the field of computer vision, computer graphics, and multimedia. A large number of salient object detection (SOD) methods have been devised to effectively mimic the capability of the human visual system to detect the salient regions in images. These methods can be broadly categorized into two categories based on their feature engineering mechanism: conventional or deep learning-based. In this survey, most of the influential advances in image-based SOD from both conventional as well as deep learning-based categories have been reviewed in detail. Relevant saliency modeling trends with key issues, core techniques, and the scope for future research work have been discussed in the context of difficulties often faced in salient object detection. Results are presented for various challenging cases for some large-scale public datasets. Different metrics considered for assessment of the performance of state-of-the-art salient object detection models are also covered. Some future directions for SOD are presented towards end.

## 1. Introduction

Salient object detection (SOD) is an important computer vision task aimed at precise detection and segmentation of visually distinctive image regions from the perspective of the human visual system (HVS). The behavior of SOD models is expected to mimic the pre-attentive stage of HVS which guides human attention to the highly interesting regions in the scene. The identified salient regions in images can facilitate subsequent high-level vision tasks for improved efficiency and optimal resource usage. As a preprocessing step, SOD has served many computer vision tasks such as, visual tracking [1,2], image captioning [3], image/video segmentation [4,5,6], and so forth.

**Figure 1:**
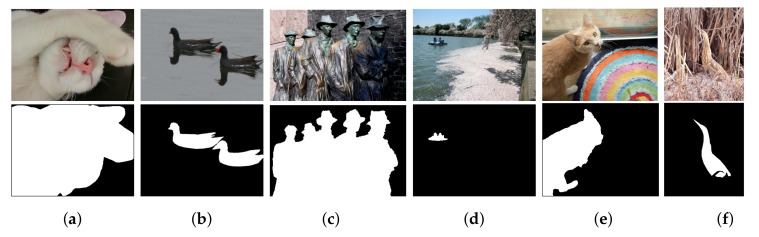
Sample challenging images for salient object detection with corresponding pixel-wise annotations shown below. (**a**) Large object, (**b**) Reflection, (**c**) Multiple objects, (**d**) Small object, (**e**) Complex scene, and (**f**) Low contrast.

The challenges and difficulties in SOD come from the very nature of the scenes captured in free viewing conditions. Several sample images from different SOD datasets can be seen in Figure 1. The accompanying pixel-wise annotations shown here are used for evaluation but clearly delineate the basic requirements for a salient object detector. A SOD method should keep the error metric values to their least by strictly attaining to the salient regions and missing the non-salient ones. It is further expected that the SOD method should be computationally inexpensive in producing a high resolution saliency map for accurate salient object localization [7]. Being an active research field over the past two decades, a large number of models have been attempted to satisfy the minimum requirements for image based SOD. Early efforts for saliency detection were focused at fixation prediction [8,9]. Fixation prediction aims to attend the spatial locations where an observer may fixate within few seconds of free-viewing. SOD is different from fixation prediction as models for the former should detect and segment the entire extent of salient regions/objects in the scene. A general approach adopted by conventional SOD models to accomplish this goal is to assign high probability values to salient elements in a scene while producing a saliency map. Once detected, techniques such as thresholding can be used to segment out the whole salient object. Conventional SOD models following Itti et al. [8] attempt to capture the notion of scene rarity or uniqueness mainly by devising center-surround contrast features. Regional contrast in terms of global and local schemes have been frequently used in conventional SOD. Various complementary heuristic saliency priors have also been deployed to effectively capture the most conspicuous object regions in images. These conventional models have been proven to be efficient and effective in relatively simple scenes with a single object and/or clean background.

Many diverse datasets have surfaced in the past ten years to challenge these SOD models. The presence of multiple salient objects, heterogeneous salient objects with variations in shape, size and position, low-contrast objects, and much cluttered background in datasets are challenging issues to address while adhering to high prediction requirements of SOD. However, the recent rapid development of deep learning-based techniques in the field has been highly successful in tackling most of the aforementioned issues. Fully convolution neural networks (FCN) lies at the core of deep learning-based SOD [10]. The powerful hierarchical multi-scale feature representation of FCN has been utilized in various ways for a coarse saliency prediction and its refinement for boundary accurate saliency map in a data-driven manner. However, the conventional models for SOD have the advantage of providing real-time performance and can be applied in the wild. Meanwhile, several deep models have leveraged saliency priors to improve the representational ability of multi-layer features and to speed-up the training process. Wang et al. [11] combined saliency estimate of multiple conventional methods as the prior knowledge informative of salient regions to guide saliency detection. Chen et al. [12] utilize saliency priors as an initial prediction for saliency refinement. Zhang et. al. [13] devised a deep unsupervised saliency detection with noisy supervision from multiple conventional SODs. Simple heuristic operator such as contrast in Reference [14] has been adopted for contrast modelling of multi-scale features in References [15,16]. These adaptations suggest that despite tremendous progress and superior performance demonstrated by deep learning based SOD, the tools of conventional saliency detection can be useful for further raising the performance bar of deep models.

Inspired by these observations, this survey aims to comprehensively cover both conventional as well as deep learning based SOD models. Various aspects of SOD are thoroughly discussed. Large-scale datasets and evaluation metrics have been instrumental in promoting research in SOD. Therefore, popular SOD datasets and recent metrics used to evaluate several state-of-the-art models in SOD are also discussed. The organization of the rest of the survey is summarized as follows. Section 2 gives the motivation behind this study and contribution made by authors. Section 3 gives a brief overview of the history of SOD. Section 4 presents a review of the conventional SOD models. In Section 5, SOD models based on deep learning are discussed. Common datasets, evaluation metrics, and a discussion on qualitative and quantitative evaluation of some state-of-the-art models are briefly summarized in Section 6. Section 7 presents the future research direction for SOD. Finally, Section 8 concludes the survey.

## 2. Motivation and Contribution

The research in SOD is inherently driven by its applicability to a wide-variety of tasks from fields such as computer vision, multimedia, and robotics. SOD has been utilized in various research and practical problems including foreground annotation [17], quality assessment [18,19,20], action recognition [21,22], video summarization [23,24], image and video compression [25,26], object discovery [27], image/video segmentation [4,5,6], visual tracking [1,2,28,29], image/video retrieval [30,31], content based image retrieval [32], image editing and manipulation [33,34,35], thumbnail creation [36], photo collage [37], image retargeting [38,39,40], object detection and recognition [41,42,43], and caption generation [3]. SOD is not a new field of research, but several intriguing directions culminating from related fields have enabled great progress in this field during the last decade alone. To be specific, several variations of deep-learning methods have had a great impact on this field resulting in some state-of-the-art performances.

The motivation behind this survey is to present a comprehensive understanding of the evolution of SOD methods to the readers in a pragmatic manner. The details of the key-elements in both conventional and deep-learning methods are provided to capture the essence of motivational ideas in the field. Although existing surveys covered a large number of methods, but present too few technical details. Due to focus on the coverage of a larger number of methods, the existing surveys could not present technical details for each method [7,44,45]. Different from the existing surveys summarized as in Table 1, this survey strives to strike a balance between the coverage of relevant methods and technical details of each method.This work covers the most recent and/or impactful methods from conventional and deep learning-based SOD. Moreover, several state-of-the-art methods are evaluated with four different metrics most commonly reported by SOD methods. This gives the reader a complete snapshot of the progress in the field as it stands today. Further, the visual results are presented with emphasis on challenging cases, where even the most effective methods show a lot of variation in performance.

**Figure 2:**
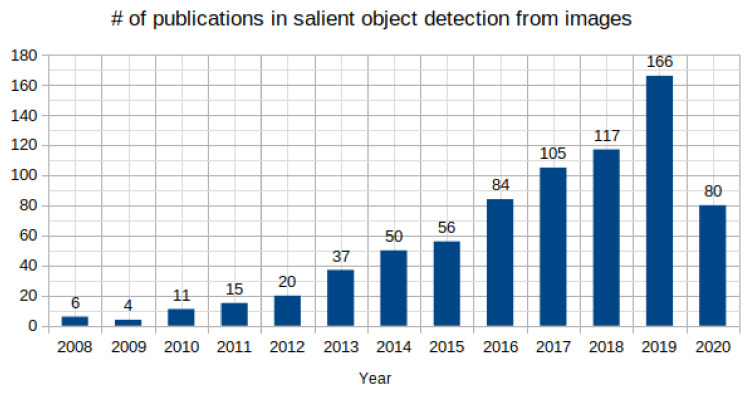
The trend of publications in salient object detection from still images from 2008–2020 (July).

**Figure 3:**
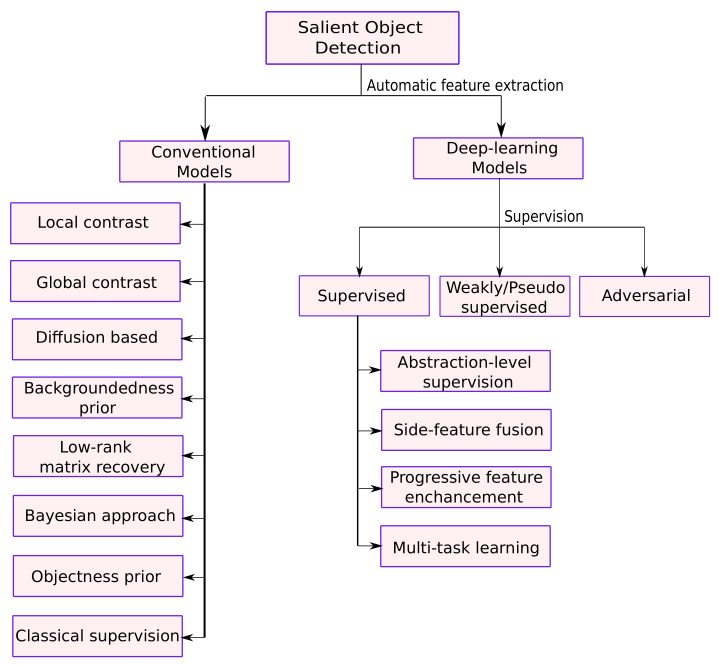
Classification of salient object detection methods used in this work.

With the intent to motivate the reader about the possible future directions in which the research could be pursued, the contributions of this work can be summarized as follows:

1. This is an attempt to cover most of the influential contributions in the past 20 years for SOD in images. Data from Google scholar advanced search with the search constraint as salient object detection from images is collected. The rise of research work as shown in Figure 2 is an indicative of the importance and usefulness of SOD in the current scenario. The present review includes 41 and 50 publications discussed from conventional and deep learning-based SOD, respectively with the aim to help readers to make a broad view of the field necessary to explore future directions for research in SOD.
2. State-of-the-art SOD models have adopted many techniques from the connected fields such as semantic segmentation. Techniques such as multi-scale contextual extraction and recurrent connections are crucial for extracting advanced features for SOD and therefore, included in this survey in a concise manner.
3. Deep learning-based SOD models are categorized based on the level of supervision during the model training. The arrangement of the most recent developments in categories of supervised, weakly-supervised, and adversarial learning is useful in understanding the key design issues of SOD models. Figure 3 presents the classification for conventional and deep learning-based methods presented in this work.

## 3. Overview of Salient Object Detection

Saliency detection has been an interdisciplinary field. The fundamental investigations on cognitive and psychological theories of HVS attention [51,52,53] were contributed by cognitive psychologists and neuroscientists. Such theories preliminarily formed the base for development of the early saliency models. A major milestone in visual saliency was achieved when the complete implementation of the computational attention architecture [53] was realized by Itti et al. [8]. The feed-forward model proposed in Reference [8] computes and combines multi-scale color contrast, intensity contrast, and orientation contrast to direct computational mechanism to highlight the salient locations in a low-resolution saliency map. Further, a winner-take-all (WTA) neural network is invoked multiple times to shift the focus of attention to the next most conspicuous location by employing inhibition of return mechanism after the first WTA invocation. This ability to shift from location to location in a fixation map is vital for tasks such as image understanding. Nevertheless, the computation of center-surround contrast using low-level features and their integration for attention guidance provided great insight for further research in the conventional SOD paradigm.

It is widely accepted that the seminal work of Liu et al. [54] and frequency tuned approach proposed in Reference [14] brought novel contributions to boost up research in SOD. Liu et al. [54] introduced the computational methods for extracting local, regional, and global features that capture different aspects of saliency information. A binary segmentation is achieved using conditional random fields (CRFs) with all extracted features. In addition to that, the first large-scale dataset was also presented in Reference [54] with bounding box annotations for training and evaluation of SOD models. Contributions by Reference [14] include in-depth frequency analysis of sub-sampled features used for contrast computation and generation of full-resolution saliency maps using a frequency-tuned approach.

Deep convolutional neural networks (CNNs) have demonstrated exceptional performance in many vision tasks such as image classification [55,56], semantic segmentation [57,58,59], object detection [60,61], and object tracking [62,63]. Deep CNNs have also benefited SOD and delivered a huge performance gain compared to the conventional SOD models. This data-driven approach generates a hierarchy of multi-scale feature representation automatically from the input image. The stacking of convolution and pooling operation in deep CNNs allows the receptive field of the network to grow gradually with depth. Due to the large receptive field, deep layers in the network could capture the global semantics and provide a holistic estimation of the salient regions. The shallow layers retain more spatial details useful for the localization of fine structures and salient object boundaries. Different deep learning-based SOD models utilize these complementary multi-layer features in various ways to learn robust saliency representations with a powerful end-to-end learning [57]. Figure 2 shows a sudden rise in the number of papers published in SOD from images since 2015 when the first few deep learning-based SOD models were proposed.

Recently, the most advanced models in SOD have been devised from the field of computer vision. Table 2 compares SOD with some related computer vision tasks such as fixation prediction [64,65], image segmentation [66,67], semantic segmentation [57,58,59], object proposals generation [68], object detection [60,61], and salient object subitizing [69]. Table 2 highlights various research tasks in the similar fields compare with SOD in terms of objective and approach taken. Although this survey focuses mainly on single RGB image based SOD models, closely related fields such as co-saliency detection(CoSOD), RGB-Depth (RGB-D) SOD, video SOD, and SOD on light field have also experienced a great deal of interest in the recent past. The CoSOD task aims at the automatic detection of the salient object(s) that are common among multiple related images. Given an image group, a co-salient object should be salient in each image along with a high chance of repeatability and appearance similarity among the related images [48]. Classical approaches to CoSOD resort to inter-image correspondence modelling strategies [70,71] to represent the common attributes among multiple images. Recent deep learning-based CoSOD models [72] learn co-salient object representations jointly, and have utilized deep-CNN models to achieve outstanding performance. Typical applications of CoSOD include collection-aware crops [73], co-segmentation [74] and video foreground detection [75]. The RGB-D based SOD models utilize important complementary information of depth along with color measurements for detecting salient objects on RGB-D images. Similar to SOD, traditional RGB-D models [76,77] rely heavily on hand-crafted features while combining RGB image with depth maps. Models [78,79] that exploit the implicit shape and contour information in depth maps to refine saliency results have shown promising performance. Deep learning-based, end-to-end RGB-D models [80,81] are becoming more and more popular as they can effectively exploit multi-modal correlations, and multi-layer information hierarchy for robust RGB-D saliency detection [82]. Video SOD models leverage the sequential, motion, and color appearance information contained in a video sequence to detect targets that are repeated, dynamic, and salient [48]. Video SOD has many applications viz., action recognition [83] and compression [84]. Very similar to other related fields, current state-of-the-art models in video SOD are deep learning-based which capture and focus on combining the spatial and temporal saliency information efficiently [74]. Efforts have also been made to deal with data insufficiency problem in the supervised video-SOD models through novel data augmentation techniques [74] or introducing new datasets [85]. The detection of saliency on 4-D light field (LF) is another interesting task related to the RGB-SOD task. A light field is an array of 2-D images which includes focal stacks, depth maps and all-focus images captured through handheld light field camera Lytro Illum [86]. In absence of a large-scale LF-SOD dataset, low-level cues have been utilized to tackle the task. Recently, Reference [87] proposed a new dataset and deep learning based model for the LF-SOD task. Interested readers may refer to References [48,82,85,87,88] for further information on these related tasks.

## 4. Conventional Salient Object Detection

The conventional SOD models elaborated in this section belong to the various advancements made in SOD before the resurgence of deep-learning techniques. These conventional models mainly exploit low-level visual features such as intensity, color, and orientation to design hand-crafted features especially useful for SOD. Most often, a salient region is considered as the part of an image that is perceptually distinct from its surroundings and thus catches the attention of a human observer. This distinctiveness, rarity, or uniqueness is widely investigated in SOD by determining the contrast of image elements to their surroundings. Methods based on contrast priors may apply local scheme, global scheme, or both to decide upon the saliency of elements in the image. As the saliency of a region can be defined in various ways, different works made varying assumptions for SOD model formulation. Priors such as backgroundness, objectness, focusness, and spatial-distribution allow the utilization of more sophisticated frameworks to attack visual saliency. Different from these low-level feature based fast approaches for SOD, the conventional supervised approach towards SOD has been exploited in many recent works [54,89,90,91,92,93]. These models are equipped with the ability to apply domain knowledge in the form of training data to saliency detection. However, these models are also dependent on the manually designed regional saliency descriptors and therefore differ from deep learning-based SOD in the feature extraction process. In this section, a thorough review of the most prominent conventional SOD methods is presented.

### 4.1. Local Contrast Based SOD

Early SOD methods determine the contrast of image elements relative to their surroundings by using one or more low-level features such as intensity, color, and orientation. The local contrast has been utilized at different levels of image abstraction such as pixels [94], patch [54], and regions [54,95]. The contrast signifies the difference among the involved elements but its varying interpretations lead to different feature representations and measures being used for distance computation.

Ma et al. [94] worked with a color-quantized CIELuv image which is sub-divided into pixel blocks. The local contrast is computed as the Gaussian weighted sum of the difference between a pixel and its local-surroundings pixels. Finally, a fuzzy-growing method is employed to segment attended points and regions from the saliency map. A parameter-free approach with simple point-wise operations such as edge detection, threshold decomposition, and the distance transform is presented in Reference [96]. Hu et al. [97] devised a linear subspace estimation method to map the 2-D image into a 1-D linear subspace after a polar transformation. The projection of all the data to the normal of their corresponding subspace considers both feature contrast as well as the geometry properties of the region. Based on this projection a new attention measure was defined.

For robustness purpose, Liu et al. [54] adapted the single scale contrast to operate at multiple scales using a pyramid. Specifically, the multi-scale contrast feature at pixels of an image is computed as a linear combination of contrasts in the L-layer Gaussian pyramid. Liu et al. [98] combined the block/pixel-based multi-scale contrast features with region information for object localization. However, the performance of this method depends heavily on the quality of image segmentation. It is also observed that pixel-based multi-contrast saliency maps emphasize high contrast edges rather than the entire salient object [54]. Further, Liu et al. [54] designed a patch-based approach for regional salient features. The $\chi^{2}$ distance of color histograms for a rectangular patch (with the area, let *A*) centred at a pixel *x* is measured from an enclosing rectangle having the same area (*A*) to find the most distinct rectangular pair at *x*. Candidates for contrast computation at a pixel are generated by varying the size and aspect ratio of rectangles in a predefined way. Different from these patch/block wise approach, Jiang et al. [95] utilized image segmentation algorithm to generate multi-scale segmentation for multi-scale local contrast. The saliency of a region at a specific scale is obtained by comparing its regional features with those of its spatial neighbors. The output pixel-wise saliency map is obtained by propagating the regional saliency values across scales to pixels.

Klein et al. [99] defined the saliency of an image region using Kullback-Leibler divergence (KLD). Specific scalable feature detectors are designed to represent the distributions in feature channels of intensity, color, and orientations. The amount of divergence in the feature statistics in the center from those in the surround is measured using KLD to estimate the center-surround contrast.

Li et al. [100] performed local contrast analysis to discover the salient regions through imbalanced max-margin learning. The local context for a centre rectangular patch includes all spatially surrounding patches that overlap with it. The inter-class separability of the center positive patch from all the surrounding negative patches is obtained from the trained cost-sensitive support vector machine (SVM). To counter the boundary imprecision in SVM saliency maps, another method based on hypergraphs is discussed in Reference [100]. The hypergraph contains image superpixels and a set of superpixel cliques as its vertices and hyperedges, respectively. The hyperegdes enforce contextual constraints on constituting superpixels due to which the problem of saliency detection reduces to reporting the salient vertices and hyperedges in the hypergraph.

### 4.2. Global Contrast Based SOD

As the local contrast operator has a limited spatial neighbourhood, large extent salient objects can be easily missed out. Further, the issues related to high-saliency at salient boundaries and low-saliency values at object interiors enable significant research towards global contrast features. Global considerations treat the similar image regions in a comparable way thereby assigning similar saliency values to uniformly highlight the entire salient region.

As an outstanding contribution, Achanta et al. [14] analysed methods such as References [8,94,101] to observe that these methods retain extremely low spatial frequency contents for contrast computation. To uniformly highlight salient objects with well-defined boundaries in a full-resolution saliency map, a frequency tuned approach was designed in Reference [14]. The method computes Euclidean distance between a Gaussian blurred version of the input image $I_{w_{hfc}}$ and the mean image feature vector $I_{\mu }$ at a pixel *x* to define saliency value as $sal\left(x\right)=∥I_{\mu }-I_{w_{hfc}}\left(x\right)∥$. This center-surround contrast computation has also inspired some recent deep-learning SOD models such as References [15,16].

Global contrast cues have been exploited in numerous SOD methods to separate a large object from its surroundings [102,103,104,105]. The computation of the global contrast for a region requires considering every other segmented region in the image. Specifically, in Reference [102] each segmented region *r* of an image *I* is represented by a color histogram. The saliency value for a region $r_{k}$ is then computed as

(1) $$\begin{array}{c} sal\left(r_{j}\right)=\sum_{j\neq k}w_{k}D_{r}\left(r_{j},r_{k}\right) \end{array}$$

where D(.,.) represents the color distance metric between $r_{j}$ and $r_{k}$. The term $w_{k}$ weights the distance between the two regions. That is, a large distance from the target region suppresses the contrast contribution and vice versa. The high global contrast leads to a better saliency value for a pixel. In a similar spirit, region uniqueness under the global contrast setting is also explored in Reference [106] using an efficient filtering based technique. With the term $∥c_{j}-c_{k}{∥}^{2}$ equated to D(j,k) in Equation (1), it is possible to effectively combine global and local contrast estimations to control the influence radius of the contrast operator, provided $w_{k}$ is a Gaussian. This is in contrast to [101,102] which perform only global contrast estimations. The parts of the decomposed version of modified Equation (1) are efficiently evaluated using a Gaussian blurring kernel on the color and squared color of the region $r_{k}$ (with $c_{k}$ as average color). Fu et al. [107] combined the color contrast and color distribution in a unified manner. Different from Reference [102], the inclusion of color distribution and distribution priors along with color contrast characteristics can better highlight the salient object(s) in complex scenarios such as strong background contrast. A saliency map refinement procedure is also presented in Reference [107] to preserve salient edge details.

To enable effective estimation of global saliency cues, large scale perceptual segments are generated in Reference [104]. This soft abstraction approach uses histogram quantization to sample appearance cues for the Gaussian Mixture Model (GMM) based decomposition. The GMM components are further clustered to get the image regions with homogenous semantic relations. These two soft abstractions allow the subsequent cues of global uniqueness and color spatial distribution to uniformly highlight entire salient object regions. Similar to Reference [104], spatial distribution prior has also been used as a complementary cue to uniqueness in Reference [54]. The prior signifies that a spatially wide distribution of a color is less expected to be a part of a salient object.

Margolin et al. [108] integrated the pattern distinctness and color uniqueness to generate the saliency map. Their patch-based statistical analysis suggested that the distance of a patch from the average patch, computed along the principal components of the image, is a robust measure of patch distinctness. Statistically, a longer accumulated such path contributes to high patch distinctness. On the other hand, global regional color contrast defines the color uniqueness of regions as a complementary cue to patch distinctness.

### 4.3. Diffusion Based SOD

The diffusion-based SOD models construct a graph structure on the image and utilize a diffusion matrix to propagate seed saliency values to the whole region of interest. Existing SOD models differ in stages like graph construction, foreground/background seed generation, and/or regulating the diffusion process. In a patch-based approach, Gopalakrishnan et al. [109] utilized the equilibrium distribution of the ergodic Markov chains on a complete graph and a k-regular graph. This generated the most salient seed and several background seeds as partial labelling on the “pop-out” graph. Finally, the labels of the unlabelled nodes were inferred using a semi-supervised learning technique. Some SOD models assume that a thin border, made-up of superpixels from the image truly signifies the background regions. These so-called pseudo-background regions are far away from the image center and therefore, work as background seeds. The Background prior based diffusion models such as References [110,111,112] first organize superpixels in the form of an undirected weighted graph where spatial smoothness and geodesic distance constraints are explicitly enforced. In Reference [110], manifold ranking is incorporated as the saliency measure to rank the similarity of image superpixels in a two-stage scheme. In the first stage, four separate regional saliency maps are computed, which reflect the relevance of the constituent superpixels to the individual side of the pseudo-background. These maps are integrated for an initial saliency map. In the second stage, the foreground nodes obtained from adaptive thresholding of the inverted initial saliency map act as the salient queries and the manifold ranking is re-applied to compute the final saliency scores for each superpixel. Filali et al. [113] have extended the formulation single-layer manifold ranking framework to multi-layer saliency graphs, and utilized texture cues along with color to more accurately detect the boundaries of salient objects.

Saliency detection via properties of absorbing Markov chains is explored in methods such as References [111,112,114]. The basic idea is to treat image boundary superpixels as absorbing nodes and the remaining superpixels as transient nodes. In this configuration, the absorption time of a transient node reflects its global similarity to pseudo-background nodes and thus, provides an estimate of saliency scores for transient superpixels [112]. To effectively suppress the long-range background regions near the image centre, Zhang et al. [111] learned a transition probability matrix. It does so by computing multiple sparse affinities with different feature layers from a pre-trained FCN-network [10] and then, infers a full affinity matrix through iterative optimization. Different from Reference [111], Sun et al. [114] identified the salient regions in an image by computing the Markov absorption probability, which represents the probability of a transient node being absorbed by an absorbing node. In a two-stage scheme, the first stage considers the background nodes as absorbing. Whereas the next stage performs a ranking-based refinement by regarding the adaptively thresholded salient nodes from the first stage saliency map as absorbing nodes. However, the performance of various diffusion methods discussed in this subsection is sensitive to specific feature spaces and scales used for the diffusion matrix definition. Very recently, a super diffusion framework [115] integrated various diffusion matrices, saliency features, and seed vectors for robust and optimum performance. Further, a supervised learning strategy is adopted to determine the closed-form solution of the optimal parameters for the integration.

### 4.4. Backgroundedness Prior Based Methods

The methods in this section are not based on diffusion but use the pseudo-background as a prior to perform SOD heuristically. Li et al. [116] built a background template B from pseudo-background to estimate the dense and sparse reconstruction errors for saliency detection. At one hand, the region-wise dense reconstruction error is computed based on the principal component analysis (PCA) bases of B which can accurately handle object segments at scene boundaries. On the other hand, sparse reconstruction error that is used to better suppress background is computed as the difference between a region and its sparse representation w.r.t the bases B. Two pixel-wise saliency maps are formed by separately integrating dense and sparse reconstruction errors at multiple scales. Finally, these complimentary maps are fused by Bayesian inference.

Images with salient objects in touch with the image border are some of the hard cases for background prior based SOD methods. Instead of assuming a pseudo-background region, the method in References [117,118] declared a patch as background when the region it belongs to is heavily connected to the image boundary. Zhu et al. [117] computed the similar regions for a superpixel *p* among *N* superpixels using the geodesic distance between *p* and all other superpixels as follows:

(2) $$\begin{array}{ccc} Area(p) & = & \sum_{i=1}^{N}exp\left(-\frac{d_{geo}^{2}\left(p,p_{i}\right)}{2\sigma_{clr}^{2}}\right)=\sum_{i=1}^{N}S\left(p,p_{i}\right), \end{array}$$

where $S\left(p,p_{i}\right)\in \left(0,1\right]$ and $\sigma_{clr}$ is set to 10. The $d_{geo}$ between any two nodes (superpixels) of an undirected weighted graph is defined as:

(3) $$\begin{array}{ccc} d_{geo}\left(p_{i},p_{j}\right) & = & \min_{p_{1}=p_{i},p_{2},\ldots ,p_{n}=p_{j}}\sum_{s=1}^{n-1}d_{app}\left(p_{s},p_{s+1}\right), \end{array}$$

where $d_{app}$ denotes the color distance in the CIE-Lab color space. The boundary connectivity for a superpixel *p* is computed as $\frac{\sum_{i=1}^{N}S\left(p,p_{i}\right)\;\delta \left(p_{i}\in B\right)}{\sqrt{Area(p)}}$ where numerator represents the length of *p* along the boundary, B. Based on these quantities, two complementary maps representing background probabilities and enhanced contrast information are generated which are further fused using an optimization framework for the final saliency map. This method is motivated by Reference [119] where Wei et al. utilized only the geodesic distances (Equation (3)) between regions and pseudo-background to assign saliency values to regions. Gong et al. [120] employed a two-stage framework for propagating saliency from simple to ambiguous regions in the image. In the first stage, a coarse saliency map is estimated from a backgroundedness prior based SOD model such as in Reference [119] and a convex hull computed on the interest points in the image. The refinement process in the second stage propagates saliency to difficult image regions using teaching-to-learn, and learn-to-teach frameworks. The real-time saliency detection methods such as in References [121,122] consider the over-segmentation as a performance bottleneck. Zhang et al. [121] designed an efficient pixel-wise raster-scanning algorithm to apply a minimum barrier distance [123] for SOD. Tu et al. [122] leveraged the minimum spanning tree representation to reveal object geometry in the scene and thus, reduced the search space of the shortest paths for the target seeds. The distance transforms in References [121,122] are shown to be robust than the distance in Reference [119].

### 4.5. Low Rank Based SOD

Several SOD models [118,124,125,126] utilize low-rank matrix recovery (LR) theory by modelling an image as a combination of two parts: a highly redundant non-salient part and a residual salient part. With a low-rank feature matrix approximating the redundant background part in some feature space, potions that deviate from the low-rank subspace are noises corresponding to the salient regions in the input image. Formally, an input image *I* is first partitioned into *N* superpixels. A *D*-dimensional feature vector $f_{i}\in R^{D}$ per region is then extracted and stacked into a feature matrix $F=\left[f_{1},f_{2},\ldots ,f_{N}\right]\in R^{D\times N}$ representing *I* in the feature space. Various LR based SOD methods mainly differ in modelling the decomposition of F into a low rank matrix $L\in R^{N\times D}$ and a sparse matrix $S\in R^{N\times D}$. A general objective function [126] for optimization can be written as:

(4) $$\begin{array}{c} \min_{L,S}\;\Psi \left(L\right)+\alpha \Omega \left(s\right)+\beta \Theta \left(L,S\right)\;s.t.\;F=L+S, \end{array}$$

where the low-rank constraint Φ(.), the sparsity regularizer Ω(.) and interactive regularizer Θ(.) take on different forms for different LR-based methods as shown in Table 3. The final saliency of *i*th superpixel is computed from the sparse matrix S as either $∥s_{i}{∥}_{2}$ or $∥s_{i}{∥}_{1}$ with $s_{i}\in R^{D\times 1}$. The Unified-LR (ULR) model [124] learned a feature transformation to effectively integrate low-level features with high-level priors. The lack of spatial relation modelling among image patches in ULR may lead to non-uniformly highlighted salient regions. The segmentation-driven LR(SLR) model [118] addressed this issue by weighing regions touching the image borders appropriately using a bottom-up segmentation prior $H_{c}$. Low-rank structured sparse matrix decomposition (LSMD) [125] and its successor structured matrix decomposition (SMD) [126] introduced a tree-structured sparsity inducing norm to capture spatial contiguity in image structures through hierarchical image segmentations. SMD further incorporates a Laplacian sparsity regularizer to help wide subspaces induced by L and S.

### 4.6. Bayesian Approach Based SOD

Given an input image, the Bayesian inference problem for saliency detection is to estimate the posterior probability of being salient at each image pixel. Xie et al. [127] estimated a convex hull based on interest points that is very crucial in estimating saliency priors and likelihood functions. The pixel specific saliency prior is computed as the fraction of its encompassing cluster that is in the intersection with the convex hull. Few such encompassing clusters are generated by grouping superpixels into larger regions using a clustering technique. The likelihood probability computation in Reference [127] is center-surround based with the convex hull and its complement representing the foreground and background region, respectively. Sun et al. [128] computed the prior map similar to Reference [127] but weights convex hull at its superpixel boundaries using probability scores for boundaries [129] and the color difference between superpixel and the background region. To improve likelihood estimation, the convex hull estimation is further refined with soft-segmentation techniques like Kernel density estimation and independent component analysis with reference (ICA-R). Wang et al. [130] utilized a fully connected CRF to infer more precise initial saliency for better likelihood computation in a geodesic weighted Bayesian framework. The method utilizes a saliency map of existing methods as the prior distribution.

### 4.7. Objectness Prior Based SOD

SOD methods based on objectness leverage the likelihood of an image window containing an object which is provided by an object proposal algorithm such as Reference [68]. Chang et al. [131] jointly estimated the objectness of potential object windows and the regional saliency by iteratively minimizing an energy function. The energy function has one term each under the explicit influence of objectness and saliency, and a third term modelling their interaction. Jia and Hen [132] utilized the objectness scores as the saliency before suppress weight values corresponding to pixels that are less likely to be foreground. This improves the influence of the foreground pixels in propagating the saliency information than background pixels in a Gaussian MRF. Li et al. [133] incorporated foreground labels obtained from the objectness measure with boundary cues in a co-transduction framework to generate improved saliency maps in complex images. In Reference [92], the priors for focusness and objectness are explored and non-linearly integrated at pixel-level with uniqueness cues to improve SOD. Generally, the salient objects in a scene receive high visual attention because of being photographed in focus. By defining the focusness as an inverse of blurriness, Reference [92] modelled it as the convolution of a sharp image with a point spread function approximated by a Gaussian kernel. The edge scales (σ in Gaussian kernel) are estimated using a scale-space analysis for estimating the pixel-level focusness. The focusness at the boundary and interior edges are propagated to compute regional focusness values. For pixel-wise objectness prior, object generation methods such as Reference [68] is utilized. To assign objectness scores at regional-level, Reference [92] computes the mean value of objectness scores of constituting pixels.

### 4.8. Classical Supervised SOD

Several supervised models based on classical machine learning (ML) algorithms have also been proposed for SOD. These methods generally contain three major steps for inference after segmenting an image into image regions. Firstly, a set of sophisticated features are manually extracted from each image region (superpixel/patch) to form its regional descriptor. Secondly, a trained linear/non-linear ML regressor/classifier predicts the saliency score/confidence from the input regional descriptor. Finally, saliency score of each region is assigned to its contained pixels for an initial saliency map. Liu et al. [54] designed a set of salient features viz., local multi-scale contrast, regional center–surround histogram distance, and global color spatial distribution to define a generic salient object. These salient features are combined through CRF learning with bounding-box annotations of the salient objects. Compared to saliency specific features in Reference [54], Mehrani and Veksler [89] utilized standard features such as color, location, size, and texture to form regional descriptors. The initial segmentation from the trained boosted-decision trees classifier is further refined with binary graph-cut optimization for accurate boundaries. Lu et al. [134] combined pre-attentive saliency maps and mid-level features for object perception and learned seeds through a large-margin framework. Kim et al. [90] represented a saliency map as a linear combination of high-dimensional color space. The high-dimensional color transform is applied on an initial saliency map estimated via a random forest regressor [135]. Mid-level cues of location, color histogram and contrast, texture, and shapes were used for regional descriptors. Wang et al. [91] formulated SOD as a multi-instance learning problem (MIL). Four different MIL classifiers are independently trained with regional feature descriptors comprising of low-level, mid-level, and boundary cues. Jiang et al. [92] utilized the regional descriptors such as local contrast, backgroundedness, and generic properties for segments from multi-level image segmentations. A random forest regressor is learned to map the regional feature vector to a saliency score for the regions. The saliency maps across multi-level segmentations are fused for the final saliency map. Yang et al. [93] developed a max-margin approach to jointly learn the CRF and a discriminative dictionary for SOD. The designed CRF model is layered in which target variables are conditioned on an intermediate layer of sparse codes of image patches. The intermediate layer assists both the CRF and the dictionary in learning.

The conventional approach to saliency detection is mainly characterized by the use of low-level feature and being fast in processing. A crucial decision regarding the performance of a method is the selection of processing abstraction. Saliency computation based on pixels/patches usually highlights high-contrast edges and misses the interior of salient objects. On the other hand, methods that adopt regions as processing abstractions are generally efficient with the ability to utilize richer feature representations for saliency detection. Further, the use of single-scale and multi-scale segmentation is related to the trade-off between efficiency and robustness.

As discussed in this section, conventional supervised approaches for saliency detection can not extract the features informative for saliency detection automatically. However, with the availability of adequate training datasets, classifier/regressor can be trained to automatically integrate a large but fixed number of regional features for the most discriminative ones. Although the performance of such learning based models is superior to their heuristic counterparts, the advances in conventional saliency detection still fall short in accurately handling saliency detection in challenging scenarios.

## 5. Deep Learning-Based Salient Object Detection

The success of training a deep convolutional neural network (CNN) [55] on large scale object recognition dataset [136] has had a huge impact on the entire research community. Researchers from diverse fields such as natural language processing [3,137], computer networks [138,139,140], stock market analysis [141,142], document analysis and recognition [143,144], and of course computer vision [55,56,57,58,59,60,61,62,63] have effectively leveraged deep-learning to devise models that achieve appreciable performance compared to heuristic, and classical machine learning (ML) techniques. Following other related fields in computer vision, existing pre-trained CNNs [56,145] for image classification task on ImageNet dataset have been re-purposed to effectively address various challenges present in the SOD task. More specifically, most deep learning-based SOD models utilize a pre-trained backbone architecture which is then fine-tuned on a small-scale SOD training dataset. In this way, the saliency detection task is benefited by reusing the semantic visual knowledge already learned in CNNs. The deeper architecture of CNNs can learn illustrative and differentiable features at multiple levels of feature hierarchy. Deep learning-based SOD models utilize this multi-level hierarchy, and introduce architectural novelties in the network to produce representations that are vital for saliency detection. These advanced multi-layer features allow the deep learning-based SOD models to capture image regions with high saliency value at coarse scale automatically. At the same time, the shallow layers in the hierarchy provide detailed information useful to locate boundaries and fine structures of the salient object(s). The multi-faceted nature of the CNNs have made them a handy tool for researches to design novel models for the SOD problem.

**Figure 4:**
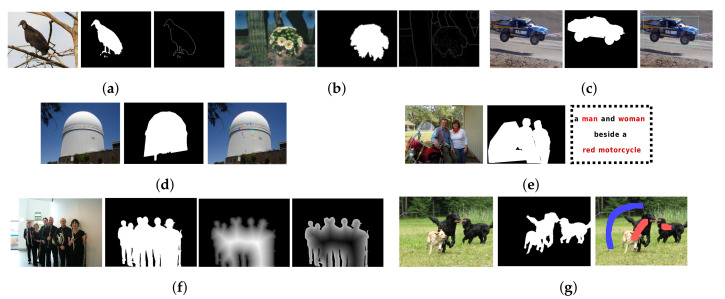
Various supervisions signals for salient object detection (From left to right in all sub-images): RGB Image and its pixel-accurate annotation followed by (**a**) boundary/contour map extracted from object-level ground-truth and used for supervision in the SOD task [146], (**b**) edge-based ground-truth used in the Edge Detection (auxiliary) task in Reference [146], (**c**) bounding-box annotations from Reference [110], (**d**) fixation annotation from Reference [110], (**e**) image caption generated by Reference [147], (**f**) example of a body-map and a detail-map obtained from the object-level saliency ground-truth of the corresponding RGB image in Reference [148] for supervising edge and interior perception respectively, and (**g**) scribble annotations are utilized for weakly-supervised SOD in Reference [149] (red marks foreground and blue marks background).

In this section, an extensive review of deep learning-based SOD models is systematically presented. The models are broadly categorized based on the level of supervision into fully supervised, weakly/pseudo-supervised, and adversarial models. Models satisfying these broad criteria are further grouped together based on the most prominent/resembling properties, common issues addressed, and/or similar architectural design. Figure 4 shows different levels of supervisions used for SOD.

### 5.1. Supervised Models

Fully supervised SOD models are designed with an implicit assumption that sufficient human-annotated training data are available. The training data includes the original images and their corresponding pixel-wise human-annotated salient object masks.

#### 5.1.1. Abstraction-Level Supervision Based

Early efforts in deep learning-based SOD models were mainly focused at utilizing deep CNN based features to predict a saliency score for each processing abstraction. Processing abstractions could be superpixels/patches [150,151] or object regions [152,153]. For training the networks, thousands of processing abstractions are extracted from the training datasets. These processing abstractions are assigned binary labels individually based on the normalized overlap ratio between a target abstraction and its ground truth saliency map [152,154]. Table 4 summarizes some representative methods in this category.

Zhao et al. [150] explored multi-context deep features for SOD. Two similar-structure CNNs are used to independently model the global and local contexts for each image super-pixel. Each CNN accepts a fixed scale window centered at the queried superpixel to define the scope of context. The extracted multi-context features for a queried superpixel are combined and regressed for a final saliency score using a shared multi-layer perceptron (MLP).

Some methods resort to a pre-trained image classification network for extracting deep features for superpixels [151,154]. Lee at al. [151] created feature descriptors for each superpixel by integrating an encoded low-level distance map (ELD-Map) with semantically stronger deep-CNN features. The ELD-Map encodes the similarities/dissimilarities between the queried superpixel and all others. An initial stack of hand-crafted feature distance maps captures such relationships for a queried superpixel, which is then processed using a simple CNN to generate the ELD-map.

Li et al. [154] exploited the multi-scale deep features to predict a saliency map. For each queried segmentation, deep features that are extracted from three different scales are concatenated and fed into a stack of fully connected layers to infer the saliency score.

In contrast to superpixel/patch, the region proposals reduce the search space by allowing the model to focus on the interesting regions where objects are likely to appear. Wang et al. [152] integrated pixel-wise local estimate with the object-aware global search for robust saliency detection. Firstly, a patch-input based deep CNN is trained to assign a saliency value to each image pixel. This local saliency estimation is further refined to include only those object proposals [155] which have high accuracy score and large coverage area w.r.t. the initial saliency map. Finally, each candidate object region is represented by a vector combining features of global contrast, geometric information, and local saliency measurements, which is processed using an MLP for a final saliency score.

Zhang et al. [153] filtered a set of scored bounding box proposals into a compact subset of detections using maximum a posteriori (MAP)-based subset optimization formulation. The method utilizes a CNN model to generate a fixed number of scored location proposals for the MAP-based optimizer.

Kim et al. [156] leveraged CNN as a multi-label classifier to estimate the closeness of a region proposal from each of the pre-defined shape classes with fixed binary representation. The final saliency of an image pixel is derived by averaging the prediction results of all-region proposals containing it.

**Table 4:** Abstraction-level supervision based models.

Method	Publ.	Year	Backbone	Training Dataset	Strategy
Multi-Context Deep	CVPR	2015	GoogleNet	MSRA10K [54]	Superpixel centered deep local
Learning (MCDL) [150]					and global context extraction.
Encoded Low Level	CVPR	2016	VGGNet	MSRA10K [54]	Learned local deep features
Distance (ELD) [151]					from heuristic ones.
Multi-scale Deep	CVPR	2017	VGGNet	MSRA-B [54] + HKU-IS [154]	Learned deep features at various scales.
Features (MDF) [154]				+ ILSO [157]	
Local Estimate-Global	CVPR	2015	-	MSRA-B [54] + PASCAL-S [158]	Refined local estimate within object
Search (LEGS) [152]					proposals, search globally for best proposals.
Maximum-a posteriori (MAP) [153]	CVPR	2016	VGGNet	SOS [69]	Optimized a set of bounding-box proposals.
Shape Saliency Detector (SDD) [156]	ECCV	2016	AlexNet	MSRA-B [54]	Utilized pre-defined shapes.

The models discussed in this sub-section mostly utilize a classification network to assign saliency scores to image elements. With features extracted from a deep stack of convolution operations, these methods outperform the various conventional method presented in Section 4. However, these models perform an element-by-element scanning of the input image for producing a saliency map, which is computationally expensive and time-consuming. Besides, the fully-connected architecture of MLP-based regressor can not preserve the spatial information from CNN features. Moreover, direct training with binary supervision at superpixel level requires handling issues related to weakly-supervised learning.

The models presented in subsequent subsections are all based on the seminal work of Long et al. [10] on fully convolutional networks (FCN). The most revolutionary step was to remove the fully connected layer in CNN and to make all learnable layers in FCN convolutional. FCN enables end-to-end learning of networks with full saliency mask based supervision. Further, the saliency inference also becomes efficient with only a feed-forward path required to generate the entire saliency map. Table 5 presents some popular classification models that are adopted by the recent SOD models as a network backbone. Among these architectures, VGGNet [56] and ResNet [145] are tremendously used in the literature.

#### 5.1.2. Side-Feature Fusion Based Models

CNNs are designed to produce multi-level feature maps through repeated pooling and stride convolutions. These operations gradually form larger receptive fields in deep layers of the feature hierarchy. Due to which features in deeper layers possess high semantic-awareness but loose most spatial-details. On the other hand, shallow-level features are rich in spatial details but are short of global information. A dense-prediction task such as SOD can benefit from multi-level features by exploring ways to convert them into more advanced representations via feature fusion strategy. The enhanced side-outputs after feature fusion are connected to the corresponding levels in the decoder or processed individually. The models discussed in this section are summarized in Table 6.

Hou et al. [167] extended the HED edge detector [168] for SOD via the short-connection strategy. Specifically, several short connections are introduced from the side-outputs of deeper layers to that of shallower ones. The enhanced multi-level features are converted into corresponding saliency maps, all of which are deeply supervised. Such deep-to-shallow messages benefit shallower side-output layers in both, locating the most salient region and learning-rich low-level features for refinement of irregular deeper predictions. Lastly, all saliency maps are fused in a weighted-way to produce the final saliency map. Fu et al. [169] crafted a unified framework called Deepside that is deeply supervised to incorporate hierarchical CNN features. The framework incorporated deeper side structures with different depths to imitate the behavior of “skip-layer” [57], “top-down” [157], and “short-connection” [167] architectures in deep learning. The deeply-supervised advanced side-outputs are further fused using a segmentation based pooling mechanism to detect salient objects with accurate boundary.

In Reference [15], Luo et al. harnessed the hierarchical structure of encoder to extract local multiscale contrast and global context features. The global image context is captured with a stack of convolutions appended on the top-most layer of the encoder. For local saliency estimation, contrast features $X_{i}^{c}$ are additionally extracted from side-output features $X_{i}$ using $X_{i}^{c}=X_{i}-AvgPool\left(X_{i}\right)$ where average pooling kernel size is set to 3×3. These discriminative features from the multi-layer hierarchy are gradually fused in a coarse-to-fine manner to generate the required local saliency features. Recently, Tu et al. [170] incorporate edge guidance in the framework designed in Reference [15]. Especially, the side features are first transformed into edge-aware features with parameters learned from coarse edge features, which are produced by a condition network with edge map as input.

To utilize the multi-scale features, some approaches [171,172] aggregate the features from multiple layers in a densely-connected manner or follow a heuristic style. Zhang et al. [171] deployed multiple shrink-and-extend modules to aggregate multi-level features into multiple resolutions. Under a deep supervision strategy, these multiple resolution predictions are hierarchy and progressively refined to support the bi-directional message passing. Each aggregated prediction is made boundary-aware before being fused for a final saliency map. Different from Reference [171], Hu et al. [172] integrated multi-level features only at a single resolution. The compressed integrated features are again merged with the features of each layer to generate their refined versions. This process is repeated several times with the successively refined multi-layer features to suppress noise in shallow-layers and promote saliency details in deep-layers features.

A computationally efficient approach in Reference [173] utilized partial decoder that fuses only the deep-layer features to generate a saliency map. After typical fine-scale layers in the encoder, the architecture forks into two branches. The partial decoder in the first branch integrates its deep features to generate an initial saliency map. This map is further made robust before being fused with the features that form the input to the second branch. This feature fusion has shown to refine the features in the backbone. The final saliency map is produced by the partial decoder of the second branch. Both branches are supervised by pixel-wise saliency annotations.

**Table 6:** Summary of Side-feature fusion based models.

Method	Publ.	Year	Backbone	Training Dataset	Strategy
Deeply Supervised Saliency (DSS) [167]	CVPR	2017	VGGNet	MSRA-B [54] + HKU-IS [154]	Introduced short connections.
Non-Local Deep features (NLDF) [15]	CVPR	2017	VGGNet	MSRA-B [54]	Extracted contrast based features.
Aggregate Multi-level (Amulet) [171]	ICCV	2017	VGGNet	MSRA10K [54]	Aggregated multi-level features into multiple resolution.
Recurrently Aggregated Deep (RADF) [172]	AAAI	2018	VGGNet	MSRA10K [54]	Performed aggregation at image resolution and propagate back.
Cascaded Partial Decoder (CPD) [173]	CVPR	2019	ResNet50	DUTS [174]	Partial decoders for computational efficiency.
Deepside [169]	Neurocomputing	2019	CGG	MSRA-B [54] + DUTS [174]	A general framework emphasizing side structures with different depths
Sub-region Dilated Block (SRDBNet) [164]	ITCSVT	2020	DenseNet	DUTS [174]	Introduced parallel-ASPP for context extraction.

**Figure 5:**
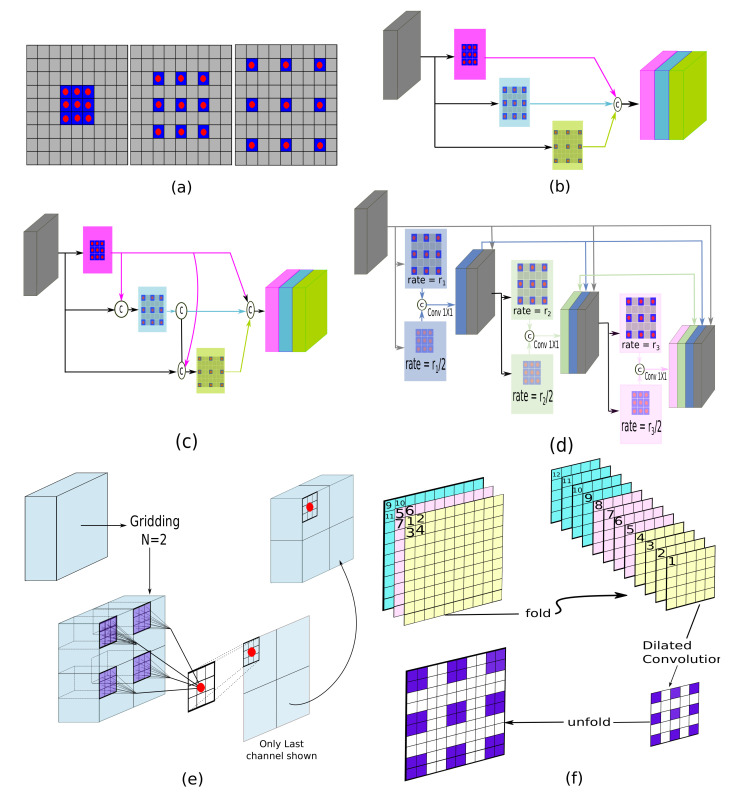
Variants of Atrous Spatial pyramid pooling module (ASPP) for multi-scale feature representation. (**a**) Dilated/Atrous convolutions with kernel 3×3 and different dilation rate of 1, 2, and 3 from left to right; (**b**) Multiple parallel dilated convolutions with different dilation rates form ASPP [58], adopted in Reference [159] for SOD; (**c**) DenseASPP module [175] connects dilation layers more densely; used in SOD models such as Reference [176]; (**d**) Parallel ASPP [164] configuration uses dilation rates of $d_{i}$ and $\frac{d_{i}}{2}$ at depth *i*; (**e**) Global perception module in Reference [177] promotes local patterns with global information; and (**f**) Fold-ASPP [166] sequentially performs fold, dilated convolution and unfold operations on input feature maps to address gridding issue in ASPP.

The atrous convolution [58] shown in Figure 5a have the advantage of enlarging the view of receptive field to extract large-scale features. This comes at no extra computational cost. Such convolution operations have been explored in various ways in SOD models for multi-scale feature extraction, see Figure 5. Recently, Wang et al. [164] fused the local contexts from multiple subregions of the feature map with its global contextual features for robust feature representation. To this end, a sub-region dilated block (SRDB) is designed that applies Parallel Atrous Spatial Pyramid Pooling (PASPP) (Figure 5d) to different sub-regions to extract rich context features which are further weighted with the global information of the input feature map. Multiple SRD blocks are utilized to refine the side-features of the network in a top-down manner. The enhanced side feature maps are finally fused for a saliency prediction.

#### 5.1.3. Progressive Feature Enhancement Models

The features in deeper layers of a CNN are supportive for object categorization but are not conducive to localizing the detected objects. This localization issue is closely related to generate boundary-aware and spatially consistent saliency maps. To this end, various saliency models adopt bottom-up/top-down architecture proposed in Feature Pyramid Network [178]. The bottom-up pathway is a feed-forward network that produces a rather coarse saliency estimate. On the other hand, a top-down pathway refines the coarse prediction by progressively and hierarchically absorbing fine features from lower layers. The refined saliency map at the finest resolution becomes the final saliency map. Several prominent models from this category are listed in Table 7 and discussed next.

The aggregation of side-features in a fully-connected manner [171] ignores the importance of multi-scale feature in feature hierarchy. Such fusion introduces information redundancy for most images and degrades the performance due to inaccurate information. For this problem, the use of gates as a mechanism to control the message passing is investigated in models such as References [159,166]. Zhang et al. [159] introduced a bi-directional structure for controlled message passing among features of different layers. Firstly, the multi-level features are refined with Atrous Spatial Pyramid Pooling (ASPP) [58] (Figure 5b) to capture image context at multiple scales. The bi-directional structure allows complementary information in refined multi-level features to be propagated from fine-to-coarse and coarse-to-fine layers under the control of gates. The refined multi-layer features are fused in a top-down manner for a final saliency map. To extract the full context of large salient objects, models such as Reference [176] have adopted a dense-ASPP module [175] (Figure 5c) for multi-scale feature extraction. Recently, Zhao et al. [166] utilized multi-level gates to control the message passing between scale-matching encoder-decoder blocks. The method introduced a fold-ASPP module (Figure 5f) that processes the deepest features of the backbone to produce a contextually-rich global representation. The progressive refinement pathway generates a finest saliency map by applying multi-layer gate units to respective lateral connections. Along with this decoder, another parallel decoder combines the compressed multi-layer features under the control of another set of multi-level gate units with the finest prediction produced. The two branches are combined in a residual way for a final saliency map. Recently, Pang et al. [161] addressed scale issue in SOD by enabling interaction of a layer with features of its adjacent layers only. Specifically, a mutual learning based interaction module at each resolution aggregates adjacent multi-scale features to improve feature representation for saliency. In the top-down integration pathway, intra-layer features are further exploited to strengthen multi-scale representation. The foreground-background pixels imbalance problem in saliency detection is tacked with a consistency-enhanced loss.

In Reference [177], Feng et al. performed two consecutive refinements of saliency features at every scale using the attentive feedback modules (AFM). Firstly, an initial coarse saliency map is computed to be fairly rich in spatial details along with semantics (Figure 5e). During scale-by-scale, top-down refinement, the laterally connected encoder-decoder pair (EDP) refines the input features for the first saliency map. The map is eroded and dilated to form a ternary attention map (TAM). The generated saliency features including TAM are fed into the same EDP to produce a second refinement. This dual enhancement within a scale specific AFM handles upsampling effects along with the deficiencies in the first saliency map. A boundary enhancement loss is applied at two shallow layers to segment out the fine boundaries.

**Figure 6:**
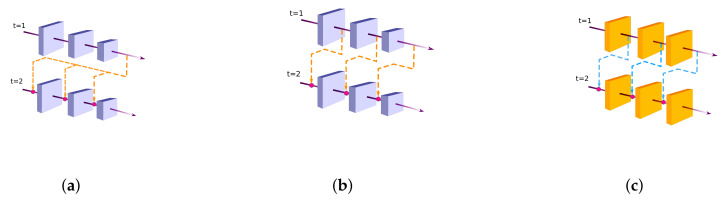
Different recurrence schemes for backbone (ResNet) network. (**a**) Multipath recurrent connections used in Reference [180]. (**b**) Blockwise recurrent scheme exploited in Reference [182]. The blue tensor indicates a convolutional block composed of many residual modules with the same scale. The orange lines represent the convolution and upsampling operations. Orange ball at t = 2 denotes elementwise addition. (**c**) Layerwise recurrent scheme used in Reference [188]. All the orange elements represent the scale-matching residual modules in a convolution block.

Several SOD approaches utilize the recurrence connections in different ways to learn useful saliency features over time. In this context, Lie et al. [179] utilized recurrent convolution layers (RCL) for refining the coarse saliency prediction. In particular, the features of a shallow layer are laterally combined with the saliency map of the previous coarse layer using the RCL. The presence of multiple recurrent connections in RCL facilitates learning, besides improve the contextual information over time. Further, all saliency maps in the top-down pathway are deeply supervised. It is noticed that the hierarchical refinement of a coarse prediction through the top-down pathway gradually dilutes the high-level features with progressive incorporation of more and more shallow features. To address this issue, Reference [180] contributed two modifications. Firstly, the multi-path recurrent connections are established from the deepest layer to all shallow layers, see Figure 6a. This way, the lower layers become aware of global semantics knowledge over time. Secondly, channel-spatial attention modules are introduced in the top-down pathway just before lateral-connections are fused. The attention based guidance allows selective fusion of multilevel contextual information and therefore, reduces false positives. Wang et al. [182] introduced a recurrent module that treats the inner blocks of the backbone as its basic recurrent units (Figure 6b). This enables the network to integrate the multi-scale contexts over time and also, provide semantic cues to lower layers for better feature refinement. The method also adopts an inception-like weighting module to selectively attend to the informative context in individual deep layers of the backbone. The resultant side-features are fused for a saliency map that is further refined for salient boundaries [189]. Recently, a multi-stage refinement model [188] incorporated recurrent connections at individual layers of the backbone as shown in Figure 6c. This model is further discussed in Section 5.1.5. In Reference [183], a pooling based model is designed to address the issue of gradual dilution of semantic features during top-down refinement. Firstly, the global guidance features (GGFs) are extracted from the deepest layer of the backbone by using the Pyramid Pooling Module (PPM) [190]. The semantically rich GGFs are appropriately upsampled for aggregation with the side-features of each layer in the hierarchy. Although good for localization, the large upsampling rates will produce unwanted aliasing effects in the aggregated feature maps. To this end, pooling-based feature aggregation modules (FAM) are specially introduced in the top-down pathway while merging the feature maps at different scales.

Recent works [181,187] attempt to improve saliency detection performance by exploring different levels of contexts across all pixels in an image. In Reference [181], an attention mechanism is utilized to select locations that provide informative context at each pixel-level. The pixel-wise contexts at the global and locals level are extracted by applying Renet model on the entire image and Conv layers on a local pixel-neighbourhood, respectively. Weights for softly attending the global and local contexts, individually are obtained via softmax normalization. In the adopted U-Net architecture for SOD, global PiCA and local PiCA are specifically used to laterally connect respectively, the deeper and the shallow level features while performing the top-down fusion. Most recently, Hu et al. [187] demonstrated promising results by embedded spatial attenuation context (SAC) modules to process the pyramidal feature maps from a feature pyramid network (FPN). The SAC module adopts two cascaded rounds of recurrent translations with varying attenuation factors to disperse the local image context adaptively over the whole feature maps. With *n* attenuation factors, a round of recurrent translation generates 4n aggregated spatial context feature maps which are selectively integrated using an attention mechanism. For SOD, the optimized context pyramid features are successively refined in a top-down manner for a saliency map.

Inspired by the success of residual learning [145] in fields such as object symmetry detection [191] and image deraining [192], a side-output residual learning based approach is presented in Reference [160]. The multi-layer residual learning approach assists in re-learning the boundary pixels for confident but coarse salient estimations in the top-down fusion pathway. To explore such fine structures in residuals, a reverse attention mechanism (RA) emphasizes the non-salient regions in the side-features. The RA output ($RA_{out}$) is obtained from next deep-layer confident saliency map $RA_{in}$ using $RA_{out}=1-RA_{in}$. A recent extension [12] to this work explored the role of saliency predictions from hand-crafted SOD methods as coarse saliency estimates. Qin et al. [184] appended a separate residual refinement network to process the coarse saliency map. Firstly, a deeply supervised encoder-decoder network is utilized to generate a full resolution coarse saliency map. A second encoder-decoder network with rather simple design is adopted to refine the residual of the coarse saliency map. Additional loss functions capturing image structural notion and intersection over union (IoU) based similarity are further exploited to improve the salient boundaries. Very recently, Qin [193] introduced ReSidual U-block (RSU) which replaces the plain, single-stream convolution of each stage of the encoder-decoder architecture with a U-Net like structure. This two-level nested U-structure not only captures the multi-scale features at intra-stage level but, also aggregates the multi-level features at inter-stage level efficiently. The RSU based deep network in Reference [193] is trained from scratch for SOD.

In other notable works, Wang et al. [185] proposed an iterative top-down/bottom-up saliency inference network. The alternate top-down and bottom-up processes generate successive fine-grained saliency and improved semantics for the other pathway to continue with. Further, the convolution layers in inference pathways are realized with RNN units to enable efficient intra-layer information propagation. Recently, Xu et al. [186] performed a joint refinement of multiscale features and predictions using conditional random fields(CRFs). The CRF energy function is designed to explicitly address the interaction between feature-feature, feature-prediction and prediction-prediction at specific scales. The cascade flow of a series of such CRFs performs the joint refinement in a top-down manner with the final saliency prediction being generated at highest resolution.

#### 5.1.4. Multi-Task Learning Based Models

The SOD models compiled in this section learn multiple supervised/unsupervised learning tasks simultaneously. The learning tasks that are performed simultaneously are assumed to be related to each other. Moreover, an explicit relationship between the tasks may be defined and enforced in the model architecture [162,194]. As SOD leverages the knowledge contained in the other tasks and vice versa, the generalization ability of the network to unseen scenarios gets better. Moreover, multi-task learning is also beneficial to address data insufficient problem in which the task-specific data is very limited for training a deep/shallow model. Table 8 enlist methods presented in this subsection.

Most of the existing deep learning-based methods suffer from coarse salient object boundaries. To solve this problem, several approaches explicitly model the boundaries of the salient objects. Su et al. [194] incorporated three streams in the SOD framework to address the selectivity-invariance dilemma. Firstly, an integrated successive dilation module is utilized to process the deepest layer features for learning the feature invariance at object interiors. A second stream exploits hierarchical multi-scale features for salient edge localization with boundary-GT supervision. Lastly, a third stream models the tough transition regions between the boundaries and the interior. The features from the three streams are fused into a feature mosaic map under the guidance of invariance and edge confidence maps. This map that represents the final prediction is supervised by the saliency GT. In Reference [162], multi-level features of SOD and edge detection are refined simultaneously through bidirectional interaction between the two tasks. To this end, individual multi-level features for each task is first extracted from the shared backbone. The two sets are gradually improved through cross-feature integration strategy implemented with a stack of cross refinement units (CRUs). Specifically, at *n*th CRU, the refined one level feature of one task is obtained by fusing the complementary deeper-level features of the other task from (n−1)th CRU. The refined features for the tasks are incorporated into two separate U-Nets with task-specific supervision.

To exploit the complementary between saliency and edge, Zhao et al. [199] explicitly fused the edge features with the multi-layer saliency features in the two-stage scheme. In the first stage, a U-Net architecture with varying kernel-size convolutions and non-linearities at decoders is utilized to extract saliency features at multiple scales. Further, the edges are modelled by fusing the features of a suitably selected shallow layer with that of the coarsest one in the architecture. In the next stage, the layer-wise saliency features from the decoder are integrated with the image-level edge features to generate the final set of side-output features. The extracted saliency maps from this set are merged for an overall saliency map. To leverage the benefits of highly discriminative saliency representation, Wang et al. [44] build a hierarchical attention mechanism that operates upon the multi-level features of the backbone. The hierarchical attention mechanism gradually increases the field of view to capture multi-scale contexts for fusion. These advanced multi-scale features are integrated with scale-matching edge information for salient object boundary alignment. The resultant features are progressively fused over a densely connected top-down pathway to generate the final saliency map.

In Reference [146], Wu et al. exploited multiple supervisions to train their framework for salient detection. Specifically, foreground contour detection (FC), edge detections (ED) and SOD supervision are utilized. Lateral connections in the top-down pathway are all equipped with the mutual learning module (MLM) for high-performance gain. MLMs at shallow-layers interact with their corresponding edge modules to model the correlation between FC and ED under respective deep supervisions. Further, intertwined deep supervision is adopted to model the interaction between FC and SOD during the top-down progressive feature enhancement. Very recently, Wei et al. [148] tackled the problem of imbalance in edge pixel distribution by decomposing saliency masks for improved supervisions. Specifically, a decoupling procedure decomposes the saliency mask into a body map and a detail map. Each map supervises an individual decoder branch that fuses the multi-level features extracted from the shared backbone. On one hand, a detail map that contains more edge points helps in better edge representation. On the other hand, a body map that lacks pixels near edges provides a distraction free supervision for interior object regions. Further, a feature interaction subnet enables information exchange between the two decoders.

Zhang et al. [147] leveraged image captioning as an auxiliary task to encode the semantic knowledge of salient objects. The two constituent subnets share a common backbone. The image caption subnet that is further coupled with a textual attention generator to produce the caption embedding feature vector. This vector is vital for saliency refinement. The caption embedded vector is therefore incorporated in a local-global multi-context feature extraction subnet for improved visual representations. The resultant maps contain complementary saliency cues that are fused for final saliency. The two subnets are jointly trained with a multi-task loss.

In Reference [197], Zeng et al. performed joint learning of weakly supervised semantic segmentation and SOD. The two subnets of the architecture perform on the deepest features from a shared backbone and are trained in two stages. In the first stage, the first subnet learns to generate a semantic segmentation under image-level supervision. Once trained, it is subsequently used to obtain the pseudo labels for supervising the second stage training for semantic segmentation. The saliency aggregation subnet computes the weighted sum of the segmentation masks of all classes to define a saliency map under the supervision of saliency ground-truth labels.

Subitizing is found to be beneficial for SOD as it counts the number of objects in a scene. He et al. [195] refined the predictions of the SOD subnet by using subitizing as an auxiliary task. The subitizing subnet, pre-trained for subitizing is connected to the SOD subnet by an adaptive weight layer. The SOD subnet is based on the U-Net architecture with skipped connections and hierarchical supervision. The adaptive weight layer lies between the two halves of Unet, whose weights are dynamically determined by the subitizing subnet. During network training, the two-subnets are fine-tuned in an end-to-end manner. In Reference [196], a gate mechanism [200] based skip-connection strategy is deployed. Functionally, the model performs a top-down progressive refinement of the coarsest feature maps generated from the encoder. These feature maps are supervised by a stack of ground-truth masks designed to perform the subitizing task. For saliency detection, corresponding saliency predictions are also supervised by pixel-wise saliency annotations. During top-down refinement, the advanced side-features for a layer are obtained by gating (point-wise multiplication) its features with the coarse features of the next layer immediately up in the hierarchy. Lastly, a fusion layer combines multi-scale saliency predictions to generate the final saliency map.

#### 5.1.5. Other Models

Models such as References [201,202] have utilized recurrence convolution layers (RCL) for SOD. The hidden states in RCL can be trained to capture historical information due to which more reliable and consistent inference can be obtained in the current time step. Further, the recurrent execution of convolution operation at the hidden state can effectively enlarge the receptive field of the output neurons over time, which enable long range spatial contextual dependencies to be captured [201]. Wang et al. [201] incorporated heuristic saliency priors into deep features of a recurrent fully convolutional network (RFCN). This is achieved by providing an image and its heuristic saliency map as input to the RFCN at first time step. From second time-step onwards, the fully recurrent connections enable the network to iteratively refine previous saliency maps by correcting prediction errors. Moreover, recurrent convolution layers are adopted in the architecture to enforce long range of spatial-temporal consistency. A pre-training strategy using semantic segmentation data is utilized for capturing generic representations of salient objects [11]. Kuen et al. [202] performed a sub-region based progressive refinement on a coarse saliency map generated by a convolution-deconvolution (CNN-DecNN) network. A spatial transform network iteratively selects an attentive sub-region for refinement via a shared recurrent neural network based CNN($CNN_{r}$)-DecNN. The established recurrence connections can provide context-aware features from previously attended sub-regions to enhance saliency refinement of sub-region in subsequent iterations, which is beneficial for SOD.

In rather simple encoder-decoder architecture, Zhang et al. [203] explored a reformulated dropout (R-dropout) mechanism in the encoder part to learn deep uncertain convolution features (UCF). Selective introduction of R-dropout after convolution layers acts as an uncertain ensemble of convolution features which is claimed to be element-wise probabilistic resulting in robust saliency prediction. In the decoder part, the checker-board artifacts of deconvolution operators are reduced by integrating restricted filter-size deconvolution with linear inter-convolutions while upsampling. Hu et al. [204] utilizes a deep network to generate high-semantic features on which strong energy function for level-set is defined. The initial coarse features from the backbone are processed through a guided superpixel filtering module to recover the full resolution saliency map. With a level-set defined on this map, the network is trained to learn saliency maps that minimize an approximated version of the level-set based loss function.

Several models are characterized by the presence of multiple shared/unshared CNN streams to get robust saliency features. In Reference [205], wang et al. gradually renovated finer structures through multi-stage refinement mechanism. A master feed-forward network in the first stage generates a coarse saliency map S. Subsequent stages perform gradual refinements of S by using a shared parameter network with the same structure as master but the top-most layer progressively discarded. Additionally, a context aggregation module extracts the rich contextual information from the deepest available features in each refinement stage. The detail output features from a stage are finally integrated into the preceding stage saliency map for a refined one. Recently, Feng et al. [188] extended [205] by incorporating cross-stage layer-wise recurrent connections (Figure 6c) and cross-stage channel attention module (CAM) in its design. The former helps refinement nets to learn richer local cues whereas the later contributes to making salient regions more spatially consistent. In Reference [157], Li et al. designed a Multiscale Refinement Network (MSRNet). To accurately localize objects of different scales, MSRnet processes three different scales of an input image with replicas of a refined VGG network which is repurposed to generate full resolution, two-channel probability map. Finally, an attention module jointly trained with MSRNet provides a soft weight for each spatial location at each scale to fuse three probability maps pixel-wise. For saliency detection in high-resolution images, Zeng et al. [206] refined local high-resolution details under the guidance of a global network. Firstly, a network generates global semantic guidance using a downsampled input image via bottom-up/top-down pathway. Under this semantic guidance, a patch sampling method provides uncertain local patches for local-detail enhancement. A local refinement module with the same structure as the global one captures local high-resolution detail for the attended region under the guidance of global semantics. Both global semantics and locally refined patches are further fused with the input RGB image for final saliency. A summary of models presented in this sub-section is presented in Table 9.

### 5.2. Weakly-Supervised/Pseudo-Supervised

The pixel-level annotations are the prime requirement for the training of fully-supervised models. Nevertheless, the large-scale pixel-wise annotation process is very time-consuming and labor-intensive. In this context, research efforts in weakly-supervised models aim at training the network with data that requires fewer annotations. Weakly-supervised annotations such as image-level tags or scribbles are easier, fast, and less demanding. However, the performance of these models depends mainly on how the weak-supervision signals are leveraged to generate acceptable pixel-wise salient masks for training the SOD branch. In contrast to weak-supervision methods which work with accurate but limited supervision, pseudo-supervised SOD models usually have access to more information which is generally not accurate for SOD. The related papers are enlisted in Table 10.

Wang et al. [174] devised a SOD with image-level tags as the main source of supervision. Firstly the classification network is jointly trained with a foreground feature inference network(FIN) under image-level supervision. Consequently, FIN can capture salient regions of category-agnostic objects. In the second stage, the SOD subnet combines the FIN map with backbone deeper side features in a top-down scheme to generate initial saliency prediction. These saliency maps are refined by an iterative CRF for the self-training of the SOD branch.

Recently, a scribble annotation-based SOD model is presented in Reference [149]. Image labeling with scribbles is easier and fast. But boundary localization in SOD may suffer due to lack of fine details and structures in raw-scribble based supervision. To this end, an edge-detection subnet is utilized along with the SOD stream. Both subnets receive features from a common backbone. Further, a gated structure-aware loss is proposed to constrain boundary localization. The edge subnet and SOD stream are additionally applied with cross-entropy loss and its partial version [210], respectively.

The pseudo-supervised SOD models have been devised to refine pixel-wise salient masks for training the SOD branch using information such as contours [209] and noisy saliency maps [13] from heuristic models. Zhang et al. [13] leveraged the noisy saliency maps generated by unsupervised saliency models (RBD [117], DSR [116], MC [112] and HS [103]) for SOD. The network contains a saliency prediction module that adapts DeepLab network [58] with dilated convolutions for improved resolution with FC layers discarded and an adaptive noise module. The predictor works in collaboration with the noise module towards fitting the noisy saliency maps.

To utilize contours for SOD, Li et al. [209] grafted a new decoder for SOD onto the original decoder of a pre-trained contour detector [211]. The decoders for two tasks i.e, contour and SOD are cross-connected to enable consolidation of contour (C) and saliency(S) knowledge in the saliency branch. The two branches are trained in an alternate fashion with C2S and S2C procedures generating saliency masks and saliency-aware contours to train the saliency branch and contour model respectively.

### 5.3. Adversarial Training Based Models

The Generative Adversarial Networks (GANs) has gained a lot of attention from researchers in fields such as image generation [212], image super-resolution [213] and so forth, due to their potential to generate highly realistic images. A typical GAN trains a pair of networks simultaneously with first termed as the generative model and second called discriminative model. The training is more like a contest where the generator attempts to generate realistic images, while the discriminator aims to discriminate between the images from the true data distribution and those images generated by the generator. GANs has also been applied to the SOD, especially to obtain sharp boundaries in the saliency map.

In Reference [214], Cai et al. designed a dynamic matching module to make the boundaries of the salient objects accurate. Unlike order-based matching of the convolutional layers between a generator and a discriminator in original GANs, the designed module achieves the best match in the adversarial training. The model also utilizes a super-pixel based approach to fuse low-level color and texture features for regional saliency score refinement.

Tang et al. [215] devised a cascaded CNNs based generator to implicitly improve salient boundaries via adversarial learning. Specifically, the generator consist of two cascade networks, the first performs global saliency estimation and the next refine it locally. The discriminator follows the strategy of conditional GANs where the adversarial loss is introduced to enforce sharp boundaries and spatial consistency. The discriminator in this model gives the judgment for every N×N local image patch to better learn local structures in salient regions. Similarly, Reference [216] introduced a correlation layer in the discriminator of the network for local patch-based comparison between synthetic saliency map and its corresponding saliency mask.

In this section on deep learning-based SOD, various high-performing models based on different supervision information are thoroughly discussed. Initial deep-learning models such as References [150,151] utilized abstraction-level supervision to surpass the performance of conventional SOD models. However, element-by-element scan by such models adds a lot of computational overhead and fully-connected layers fail to preserve the spatial details from CNN features. Current deep learning-based models are based on fully convolutional networks [10] which allow for pixel-level supervision, high model expressivity, and end-to-end training to extract informative object representation automatically in a data-driven manner. The hierarchical multi-stage structure has separate layers encoding features for global semantics and local details. To tackle the issue of the requirement of a large dataset and its pixel-accurate ground truth for training a model, pre-trained FCN based networks [56,145] are fine-tuned to minimize cost and reuse semantic knowledge. The various models presented within different sub-categories adopt a general approach while being specific in technical novelties as described in various subsections. Repeated pooling and stride convolutions are essential operations of CNNs to capture multi-levels features. Adversely, they contribute to reduce the spatial resolution of input and make it hard to recover the detailed information to accurately detect salient boundaries. Various side-feature fusion-based and progressively refinement based models have been proposed to handle this issue. Different from other models, several models such as Reference [183] explicitly address the problem of feature dilution with progressive refinement. Context-extraction models such as Reference [181] apply computation extensive operations to produce state-of-the-art results in the field. The utilization of additional but related learning information for SOD such as edge [199], and subitizing [195] can benefit both the tasks in a multi-tasking based model. Recent weakly-supervised models utilize novel ways to learn and predict SOD with minimum efforts for dataset annotation. Despite the success of deep learning-based models in the SOD-task, many complex cases require the special attention of the community (Section 6). Furthermore, training and inference time for deep-learning is a challenging issue. Some recent models such as References [160,162,173] have shown a high improvement in inference time with fair prediction performance. Finally, the issues associated with acquiring huge training data, its pixel-accurate ground-truth, and more importantly keeping it from biases is a daunting task for deep learning-based models.

## 6. Datasets, Evaluation and Discussion

### 6.1. SOD Datasets

The introduction of new datasets for SOD in the past decade has brought new challenges and open-up novel directions for research in the field. Table 11 enlist the most popular datasets in the field of SOD. The seminal work of [54] presented a large-scale image dataset with two parts, Microsoft Research Asia **(MSRA)-A** and **MSRA-B** [54] with bounding boxes based salient object annotations. Due to issues such as inaccuracies in bounding box annotations, very few images with multiple salient objects, and dataset biases towards image center, these datasets are now rarely used for pixel-wise model evaluation. All other datasets provided in Table 11 are annotated with pixel-wise binary masks. Images in **ASD** [14] contain only one unambiguous object present in the mostly clean background which can be effectively highlighted by rather simple saliency detectors. **MSRA5k** [95] and **MSRA10k** [54] are respectively, the fully annotated versions of **MSRA-B** and 10,000 images sampled from **MSRA-A/B**, the later is a superset of ASD. **BSD-SOD** [217] is a 300 images pixel-wise annotated dataset obtained from Berkeley segmentation dataset (**BSD**) [129]. Images with multiple salient objects, low-contrast of objects to background and objects touching image-boundaries in **BSD-SOD** introduce interesting concerns for saliency models. Datasets such as Extended Complec Sceene Saliency Dataset (**CSSD**) and extended version (**ECSSD**) [103] contains respectively, 200 and 1000 semantically meaningful but structurally complex images which are acquired from BSD dataset [129], PASCAL VOC [198] and the internet. **DUT-OMRON** [110] contains 5168 images with multiple objects, high image content variations and complex backgrounds. The availability of bounding-box and pixel-wise annotations along with fixation data allows this dataset to be used for tasks such as localization and fixation prediction in addition to SOD. **PASCAL-S** [158] dataset contains 850 complex scene images from PASCAL VOC dataset [198]. As PASCAL VOC is labelled for only 20 object categories, salient objects other than these classes are not annotated in **PASCAL-S**. Among 4447 complex images in **HKU-IS**${}^{1}$ [154], the majority of images contain multiple disconnected objects distributed over the image and have low-contrast with the background. **DUTS**${}^{1}$ [174] is the latest released large-scale dataset that contains 10,553 and 5019 images in training and test sets respectively. Both training and test sets contain complicated scenes for SOD which are selected from the ImageNet [136] train/val and test set, respectively. More recently, DUTS [174] dataset has been utilized to produce prime supervision signals for SOD viz., scribble-DUTS in Reference [149], and body-/detail- map in Reference [148].

Recent datasets that promote research in SOD related fields such as instance-level segmentation and high-resolution SOD are briefly discussed next and are enlisted in Table 11. **XPIE** [220] (name based on its subsets) is a 10,000 images dataset which is divided into three subsets i.e, Set-P, Set-I, and Set-E with 625, 8799, and 576 images respectively. Pixel-wise GT annotations are provided for each image in the dataset. Furthermore, Set-I, Set-E, and Set-P are annotated with object tags, eye-fixation data, and geographic information (for places-of-interest) respectively. Instance Level Object segmentation (**ISLO**) [157] is a pixelwise salient instance annotated and coarse contour labelled dataset that contains 1000 images. These images are collected from other datasets such as References [110,154] to have high confidence over the salient object regions. Salient Object in Clutter (**SOC**) [221] dataset contains 3000 images with the presence of one or more salient object(s), and another 3000 images void of any salient object. Images with salient objects are annotated to provide instance-level supervision, and information related to object category and challenging attributes. Zeng et al. [206] have contributed **HRSOD** and **DAVIS-S** datasets, which are two high-resolution(HR) datasets to assist research in the HR-SOD task. The HRSOD dataset contains 2010 images that are divided into 1610 training images and 400 test images. For this first HR-SOD dataset, 40 subjects had contributed to annotating the pixel-level ground truths. DAVIS-S is a rather small scale dataset with a collection of 92 images from a densely annotated high-resolution video segmentation dataset knows as DAVIS [222]. The object-level pixel-wise annotations for the selected images are generated by ignoring the categories of objects in the DAVIS dataset.

From Table 11, it can be noticed that more recent datasets are the collection of images with emphasis on multiple connected/disconnected objects in natural views with the cluttered background. The recent trend is to both train and evaluate novel SOD methods on large-scale, challenging datasets such as References [110,158,174]. Regarding annotations of the salient objects in images, it is generally performed manually with specific instructions to subjects (generally more than one) on selecting salient objects in free-viewing conditions. Final annotations for individual images in the datasets may be obtained through “majority agreement” rule [54] or consistency analysis [217] among varying annotations provided by different subjects.

### 6.2. SOD Evaluation Metrics

Performance evaluation of a salient object detector requires quantifying the degree of agreement between the saliency prediction results and the ground-truth annotations for different SOD datasets. Among the various evaluation measures reviewed in this section, overlap-based evaluation measures like precision-recall (PR) and receiver operator characteristics (ROC) have been used from some very earlier works on SOD, while others like Enhance-alignment measure (E-measure) and Structural measure (S-measure) have been introduced only recently to evaluate saliency maps more comprehensively. A brief description of some standard evaluation metrics for SOD is presented next.

1. **Precision-Recall (PR)** computation demands the conversion of an input saliency map *S* into a binary map *B* for comparison with ground-truth annotation *G*:
  (5) $$\begin{array}{ccc} Precision & = & \frac{\left|B\cup G\right|}{\left|B\right|},\;Recall=\frac{\left|B\cup G\right|}{\left|G\right|} \end{array}$$
  The most popular method to binarize saliency prediction *S* into binary map *B* is to threshold *S* using a fixated range varying from 0 to 255. Based on the thresholded binary maps, 256 pairs of precision-recall values are then plotted into a precision-recall (PR) curve which serves as a situational model performance descriptor. Contrary to this, precision-recall pair can also be reported at an image-dependent adaptive threshold [14], computed as:
  (6) $$\begin{array}{ccc} Th_{adaptive} & = & \frac{2}{W\times H}\sum_{x=1}^{W}\sum_{y=1}^{H}S\left(x,y\right), \end{array}$$
  which is the double of the mean saliency computed over *S* with *W* and *H* representing the width and the height of *S*, respectively.
2. **F-measure** [14] is computed as a weighted harmonic mean of Precision and Recall:
  (7) $$\begin{array}{ccc} F_{\beta } & = & \frac{(1+\beta^{2})Precision\times Recall}{\beta^{2}Precision+Recall}, \end{array}$$
  where $\beta^{2}$ is often set to 0.3 for weighing precision more than recall. Due to the comprehensive nature of F-measure curves, they are preferred over PR-curves to compare the performance of different methods. Alternatively, the maximal$F_{\beta }$ values from the F-measure curve or the $F_{\beta }$ value at an adaptive threshold such as Equation (6) have also been reported.
3. **Receiver Operator Characteristics (ROC)** curve plotting requires computation of True positive rate (TPR) and False positive rate at all fixed threshold values in the range [0–255]. With *B* and *G* representing maps as in Equation (5), TPR and FPR can be defined as:
  (8) $$\begin{array}{c} TPR=\frac{\left|B\cup G\right|}{\left|G\right|},\;FPR=\frac{\left|B\cap G\right|}{\left|B\cap G|+\right|\bar{B}\cap \bar{G}|}, \end{array}$$
  where $\bar{B}=1-B$ and $\bar{G}=1-G$. Methods having the ROC curve closer to the upper right achieve better performance.
4. **Area under ROC curve (AUC)** is a scalar quantity calculated as the area under the plotted ROC curve. An AUC score of 1 indicates to a perfect SOD model, while a score around 0.5 indicates random saliency prediction and therefore, a high score is better.
5. **Mean Absolute Error (MAE)** [106] penalizes those SOD methods that do well in salient object regions but additionally switch-on pixels in non-salient regions. MAE computes the mean pixel-wise absolute difference between normalized continuous prediction map *S* and the binary ground truth G as
  (9) $$\begin{array}{ccc} MAE & = & \frac{1}{W\times H}\sum_{i=1}^{W}\sum_{j=1}^{H}\left|G(i,j)-S(i,j)\right|. \end{array}$$
  A smaller MAE score relates to the better performance as it reflects the high similarity between the saliency map *S* and the ground truth *G* considering all pixels in the image.
6. **Weighted $F_{\beta }$ measure** [223] resolves the flaws caused by dependency between false-negative pixels and the spatial location of false-positive pixels in computation of $F_{\beta }$ with non-binary saliency masks. The error re-weighted versions (ω) of four basic quantities TP, TN, FP, and FN are defined by incorporating foreground pixel location affinities and background pixels locations w.r.t. foreground into weighing terms. The $F_{\beta }^{\omega }$ is defined as:
  (10) $$\begin{array}{ccc} F_{\beta }^{\omega } & = & \frac{\left(1+\beta^{2}\right)Precision^{\omega }\times Recall^{\omega }}{\beta^{2}Precision^{\omega }+Recall^{\omega }}. \end{array}$$
7. **Structural measure (S-measure)** [224] addresses the shortfall of pixel-wise error based evaluation measures in capturing the structural information by favouring foreground structures in the continuous saliency map. S-measure combines structural similarities computed at region-aware $\left(S_{r}\right)$ and object-aware $\left(S_{o}\right)$ levels as:
  (11) $$\begin{array}{ccc} S & = & \alpha \times S_{o}+\left(1-\alpha \right)\times S_{r} \end{array}$$
  where α is set to 0.5.
8. **E-measure.** [225] Enhance-alignment measure is another recently proposed measure which captures the image-level statistics and local pixel matching information of a binary map in a single term named enhanced alignment matrix $\phi_{S}$ using which the measure is defined as follows:
  (12) $$\begin{array}{ccc} Q_{s} & = & \frac{1}{W\times H}\sum_{i=1}^{W}\sum_{j=1}^{H}\phi_{S}\left(i,j\right). \end{array}$$

### 6.3. Comparison and Analysis

**Figure 7:**
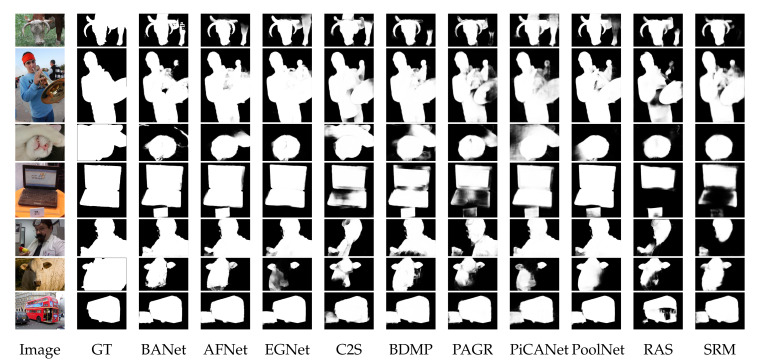
Visual comparisons of state-of-the-art on one challenging case: larger objects. Compared models are: BANet [194], AFNet [177], EGNet [199], C2S [209], BDMP [159], PAGR [180], PiCANet [181], PoolNet [183], RAS [160], SRM [205].

**Figure 8:**
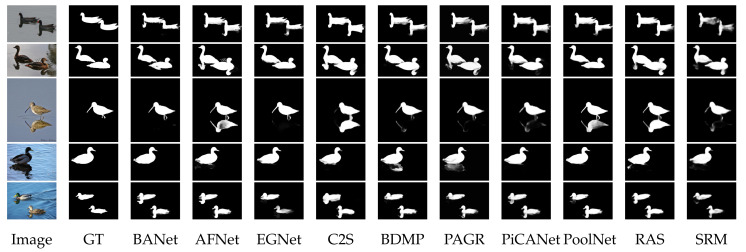
Visual comparisons of state-of-the-art on one challenging case: reflection. Compared models are: BANet [194], AFNet [177], EGNet [199], C2S [209], BDMP [159], PAGR [180], PiCANet [181], PoolNet [183], RAS [160], SRM [205].

**Figure 9:**
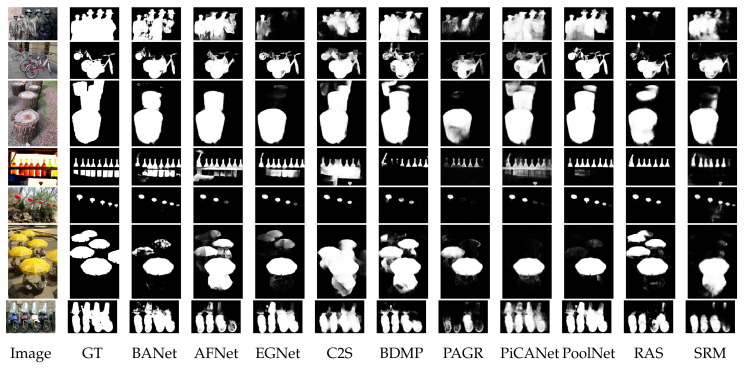
Visual comparisons of state-of-the-art on one challenging case: multiple objects. Compared models are: BANet [194], AFNet [177], EGNet [199], C2S [209], BDMP [159], PAGR [180], PiCANet [181], PoolNet [183], RAS [160], SRM [205].

**Figure 10:**
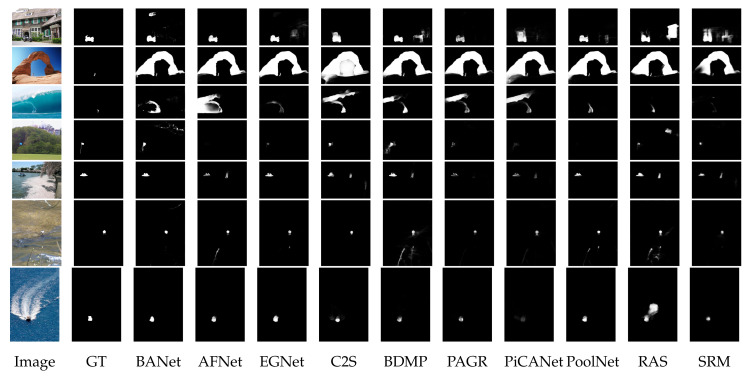
Visual comparisons of state-of-the-art on one challenging case: small objects. Compared models are: BANet [194], AFNet [177], EGNet [199], C2S [209], BDMP [159], PAGR [180], PiCANet [181], PoolNet [183], RAS [160], SRM [205].

**Figure 11:**
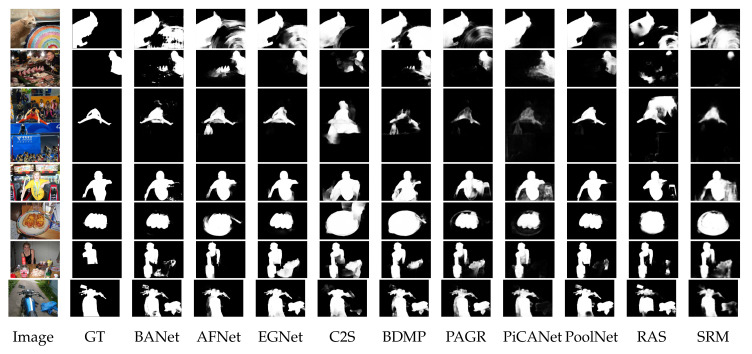
Visual comparisons of state-of-the-art on one challenging case: complex scenes. Compared models are: BANet [194], AFNet [177], EGNet [199], C2S [209], BDMP [159], PAGR [180], PiCANet [181], PoolNet [183], RAS [160], SRM [205].

In this subsection, visual and quantitative performance of some leading deep learning-based models are compared, and the related results on five most popular datasets are shown in Table 12 and Figure 7, Figure 8, Figure 9, Figure 10 and Figure 11. The results are obtained by implementing/executing the source code/algorithms provided in the respective paper.

Recent deep-learning SOD models (MINet[161], SACNet[187], GateNet [166], $U^{2}-Net$ [193], LDF [148], DSRNet [164], EGNet [199], PoolNet [183], AFNet [177], MLMS [146], PAGE [44], CPD [173], BDPM [159], JDF [186], RAS [160], PAGR [180], C2S-Net [209], PiCANet [181], DSS [167], UCF [203], MSRNet [157], ILS [174], NLDF [15], AMULet [171], SCRN [162], BANet [194], BASNet [184], CapSal [147], DGRL [182], SRM [205]) are quantitatively evaluated using four evaluation metrics on five SOD datasets (DUTS-TE [174], DUT-OMRON [110], HKU-IS [154], ECSSD [103], Pascal-S [158]). The evaluation metrics used are maximum F-measure ($maxF_{\beta }$) [14], S-measure [224], E-measure [225], and mean average error (MAE) [106]. The image-dependent adaptive thresholding method [14] is adopted to threshold the non-binary maps to compute the E-measure values. As can be seen from Table 12 that the more recent models such as SACNet [187], MINet [161], GateNet [166] and EGNet [199] are performing much better across various evaluation metrics for all five datasets. Particularly, SACNet [187] improves the MAE by 20%, 20.6%, and 8% compared to GateNet [166] on ECSSD, HKU-IS, and DUTS-TE datasets, respectively. For VGG backbone, MINet [161] gains a performance improvement by 12.1%, 14.2%, 11.3%, and 15.5% on MAE against EGNet [199] for ECSSD, HKU-IS, DUTS-TE and PASCAL-S datasets, respectively. These MAE values indicate that the recently proposed methods are able to suppress the background better while focusing on the salient regions.

The models that are included for qualitative comparison are edge/contour based SOD models (BANet [194], EGNet [199], C2SNet [209]), progressive feature enhancement models (AFNet [177], BDPM [159]), PAGR [180]), contextual attention based model (PiCANet [181]), pooling intensive model (PoolNet [183]), reverse attention based residual model (RAS [160]), and stage-wisely model (SRM [205]). Five challenging cases for SOD belonging to large objects (Figure 7), reflection (Figure 8), multiple objects (Figure 9), small objects (Figure 10), and complex objects (Figure 11) are discussed.

Figure 7 shows the qualitative performance of some recent deep SOD models on images with one large object. The detection of a large object in a scene requires additional multi-scale contextual information to cover the full extent of such salient objects. As can be seen, the RAS model that relies directly on backbone features and does not compute contextual information has suppressed many salient areas for most images. Edge-based models such as BANet [194], AFNet [177], EGNet [199], C2S [209], and PoolNet [183] are able to generate the boundary-aware saliency maps but the presence of strong edges within the large object may deteriorate their performance (rows 1, 3 and 6 in Figure 7). Additionally, a false positive similar in semantics to the salient object (rows 2 and 9 in Figure 7) is hard to suppress case during salient object refinement. Some images with salient-object and their reflections are considered in Figure 8. For this one challenging case and considered images, the performance of edge methods such as BANet [194] and EGNet [199] is better than the other models. This may be partially possible due to photographic bias which blurs the various features in the regions of reflection and the background. Another complex case is the presence of multiple objects in a scene is presented in Figure 9. The presence of multiple objects with similar semantics is a challenging issue as the number of objects and their shape, size, locations, and illumination in the scene may all be varying. Failure in the detection of significant edges and suppressing the background edges may result in missed true positives and highlighting the true negatives in edge-based models. The RAS model [160] can refine the object boundaries to highlight multiple objects (See Figure 9, row 6 ) provided it remains successful in capturing the entire semantics information properly. For small objects in scenes (see, Figure 10), issues such as appropriate detection at coarse level and feature aggregation strategies so as to avoid dis-tractors during progressive fusion are crucial. In this context, the BANet [194] applies a mosaic feature fusion strategy to learn salient pixels in the transition regions between a coarse detection and its edges. On the other hand, PoolNet [183] directly integrates the global guidance information (by upsampling) in the top-down pathway to avoid feature distraction caused by progressive feature fusion. Despite these attempts relatively large non-salient objects in contrast with the background may generate false positives, see (Figure 10, row 2). For the last two images in Figure 10, various compared models can locate the small object present in the scene but fail to capture the boundary of these small objects accurately. The fine structures such as the legs of the bird in the second last image are hardly highlighted except by AFNet [177], EGNet [199], and BDPM [159]. Moreover, some other contrast regions are wrongly highlighted by these models. The presence of humans in the boat (the last image) has not been captured by most of the compared saliency maps.The complex scenes in Figure 11 contain exactly one salient object in a cluttered background. The per-pixel local/global contextual information utilized by PiCANet [181] is useful in this scenario. Further, the prominent edges in the background may highlight distractors in edge-based models. The visual comparison among different SOD methods highlights the fact that no single model can fully handle the variety of challenges present in SOD.

**Run-time performance:** For evaluating the running time, representative methods are selected from conventional (Saliency Filters (SF) [106], Manifold Ranking MR) [110], Robust Background Detection (RBD) [117]), classical-ML-based (Discriminative Region Feature Integration (DRFI) [92]), and deep learning-based SOD models. Among these conventional SOD models, SF utilizes the low-level cues whereas MR and RBD leverage background prior in different ways for the SOD task. DRFI [92] is a high-performance heuristic model that resort to classical-ML approach to integrate a large of heuristic regional descriptors. deep learning-based SOD models belonging to different sub-categories such as abstraction-level supervision (MCDL [150]), side-feature fusion (AMULet [171], EGNet [199], CPD [173]), simple encoder-decoder enhancement (UCF [203]), context-extraction (PiCANet [181]), progressive feature refinement (RAS [160], PoolNet [183], AFNet [177], BASNet [184]), multi-tasking (SCRN [162]) and weakly-supervised SOD (C2S-Net [209]) are considered for execution-time comparison. The average run-time evaluation is conducted on a workstation with Intel Xeon(R) Bronze 3104 CPU@1.70 GHz × 12, and an Nvidia Quadro-P5000 GPU with 17 GB RAM. As shown in Table 13, the run-time of conventional SOD models is fairly high in absence of any accelerator. However, these models only exploit the low-level features and/or saliency priors which hardly capture the high-level contextual information necessary for accurate saliency detection. Therefore, even these popular conventional models score low on various saliency metrics (MAE: above 0.163 and $maxF_{\beta }$: below 0.685), and produce inferior saliency maps for complex scenarios. The classical-ML-based DRFI [92] scores lowest in the ranking as it consumes most of its time in extracting features on the multi-level segmentation in absence of an automatic feature extraction capability. Most existing deep-learning models have focused on improving the prediction performance for SOD but several models [160,173] have explicitly included techniques to address the low run-time issue. The inference time of MDCL, PiCaNet, and high performing EGNet is more as compared to other deep learning-based models in Table 13. While MDL is a super-pixel level supervised deep-learning model, the high run-time of PiCANet and EGNet is contributed by their context-extraction strategies. The PiCANet utilizes LSTM model where as EGNet deploys a set of high rate convolution kernels at multiple scales to extract useful contextual information. Models working with reduced channel dimensions such as RAS [160], SCRN [162], and BASNet [184] show high performance. The RAS model considers integrating the reversed prediction maps into the learned features for efficiency. The CPD [173] model, on the other hand, cuts-off the skip-connections of low-level features to the decoder to improve the execution time, and devised a cascaded structure to keep the detection performance high. The key to improve the efficiency of a model lies in introducing technical novelties (as in RAS [160], AFNet [177]) in the model which allow it to work with fewer channel dimensions or to discard high-resolution information (PoolNet [183]) while providing a fair prediction performance.

**Cross-dataset analysis:** The training of a deep learning-based model with different large-scale training datasets can influence the inference results i.e, the prediction performance of the model. In this context, Wu et al. [173] retrained some existing models such as NLDF [15], AMULet [171] (originally trained on MSRA10k [54]) on DUTS dataset, and observed the improvement in the performance of these models on complex scenes. The effectiveness of a dataset in generalization can be judged using the cross-dataset analysis [226]. In our survey paper, a simple network, PoolNet [183] is chosen for cross-dataset analysis in which the network is trained on two different datasets, and each trained network is tested on other evaluation datasets (DUTS-TE [174], DUT-OMRON [110], HKU-IS [154], ECSSD [103], Pascal-S [158], MSRA10k [54]). Two large-scale datasets DUTS-TR [174] and MSRA10k [54] (randomly selected 8000 images for training) are selected for training the ResNet version of PoolNet [183]. When trained with DUTS-TR dataset, the $maxF_{\beta }$ (maximum F-measure) for DUTS-TE [174] dataset and average $maxF_{\beta }$ for other datasets are 0.885 and 0.901 respectively. The MSRA10k dataset-based trained model has 0.91 as the $maxF_{\beta }$ for its training set whereas the average $maxF_{\beta }$ over other datasets is computed to be 0.85. A percentage change of −6.5%, and 1.7% is recorded from mean $maxF_{\beta }$ to the testing set of self $maxF_{\beta }$ of MSRA10k, and DUTS-TR datasets respectively. The low percentage drop of DUTS-TR dataset indicates that its generalization ability is better than the MSRA10k dataset. This result is indicative of why most recent SOD works have trained their models using DUTS-TR dataset [174].

## 7. Future Recommendations

Despite being a very active research area for the last two decades, the frequent introduction of new network architectures, better aggregation strategies and additional loss functions partially imply that future SOD networks should be able to fulfill the basic aim of SOD in most complicated scenarios. In this section, some future directions for SOD are discussed.

**Contextual information:** Context plays a significant role in detecting the full-extent of large salient objects in a scene. To extract pixel-wise local and/or global contextual information, modules such as Long- Short Term Memory (LSTM) [227] is utilized in Reference [181]. Different variants of Atrous Spatial Pyramid Pooling (ASPP) [58] and Pyramid Pooling Module (PPM) [228] are adopted in SOD models [159,183,194,205] to compute multi-scale context-aware features. Accommodation of the local neighborhood while computing global context [177] can extract a more useful per-pixel context compared to the direct application of ASPP. Although, non-local networks [229,230] can effectively model pixel-wise contextual similarities in SOD [231] but their use is limited due to huge computational cost. Recently, attenuation context based method [187] has achieved the state-of-the-art in SOD. Existing SOD methods compute local and/or global information at all image pixels in a uniform manner. However, the context demand of each pixel does vary depending on the factors including input image and its feature abstraction in the CNN hierarchy. Therefore, an adaptive procedure for context extraction may be useful in constructing an optimal context for each image pixel by identifying those image regions with high relevance to the target pixel. Such an adaptive context is expected to benefit deep SOD methods by learning the correct local/global context for each image pixel. It may also contribute towards reducing the feature interference while performing feature aggregation.

**Feature aggregation:** Many deep learning models have confronted with the issue of how to extract the effective features and aggregate them given the multi-scale and multi-level features of a pre-trained CNN network. For feature aggregation, a coarse approach which combines all-level feature into the transport layer [171] may introduce information redundancy and noisy feature interference in the model. On the other hand, exercising excessive control of information exchange between stages [159] may severely hamper the learning ability of the network. Similarly, top-down feature aggregation also requires explicit handling of aliasing effects due to large upsampling operations as done in Reference [183]. These prominent issues with the feature aggregation suggest that while merging features from different layers one should keep focus on reducing aliasing effects and noise interference to generate useful features for saliency detection.

**Loss functions:** Binary cross-entropy loss (BCEL) function is widely accepted loss criteria in deep SOD models. However, BCEL ignores the inter-pixel relationship while accumulating the per-pixel loss for a fixed batch-size. The obvious presence of multi-scale objects in SOD datasets also requires careful modelling at the loss function level to tackle the inherent fore-background imbalance problem in images. A similar imbalance problem between positive/negative classes for edges has been addressed in Reference [168] using a per-pixel weighing mechanism. Loss incorporated in Reference [11] also combines weighted cross-entropy loss with evaluation metrics to handle fore/background imbalance. Losses in References [15,177] have utilized additional loss terms such as Intersection over union (IoU) to improve boundaries of the salient object(s). Very recently, a consistency enhanced loss (CEL) for spatial coherence is proposed in Reference [161]. These efforts toward loss function design justify that a loss function targeted at general and/or specific model design issue with accompanying gradient analysis can be decisive in improving model performance.

**Inspiration from conventional models:** Few deep learning-based SOD models have incorporated saliency maps from conventional SOD models as a saliency prior to guide saliency process [160,201]. In Reference [201], saliency priors are utilized to initialize a recurrent framework, whereas a prior saliency map can replace the coarse saliency map for the reverse attention based refinement in Reference [160]. The method in Reference [15] applies average pooling to capture center-surround based contrast features as an intermediate layer inspired by a heuristic contrast operator in Reference [14]. On a different note, Reference [177] implemented the operators of dilation and erosion through max-pooling to create a turnery attention map. Exploiting different ways to integrate heuristic saliency priors or tools in a deep SOD is expected to improve both its training as well as inference.

**Dataset related issues:** The availability of large datasets with less bias is crucial for developing SOD models. The existence of bias in the training dataset hampers the generalization ability of the model to attend salient objects in complex scenarios. Existing SOD datasets can be quickly browsed to observe the presence of center bias and data selection bias. Images that are too general for salient object detectors are generally removed while collecting images for the datasets. Examples include images void of salient regions, full of cluttered background and salient object away from image borders. While keeping the scale large, it is very much essential to come up with datasets having more realistic scenarios with less bias. Another interesting trend in SOD is to show that a proposed model outperforms the ground truth on some selected images. This issue is related to annotation inconsistency at intra/inter-dataset level. To improve upon this situation more rigorous annotation procedure should be formulated and emphasis should be on fine labelling.

**Real time performance:** Very recently, DNN models [12,193] have been proposed to target the needs of mobile and embedded applications. The residual learning model in Reference [12] only learns the residual in each side-output of FCN to refine the global prediction step by step. Realizing this through a convolution layer with fewer channels results in a compact model and high efficiency. Qin et al. [193] designed a two-level nested U-structure, light-weight network which is trained from scratch for SOD. Recently, a knowledge distillation based pixel-wise saliency prediction is also proposed in Reference [232] to tackle large memory footprint issue. In computation and memory-constrained environments, it is challenging to keep the detection accuracy high with the reduction in model capacity.

## 8. Conclusions

In this work, a survey on salient object detection (SOD) from images is conducted. Among the hundreds of models presented in the last two decades from the conventional SOD and deep learning-based SOD, the most influential models and the recent advances in the field have been reviewed. Conventional models that employ low-level hand-crafted features or heuristic priors are generally efficient and effective for scenes with a single object and simple background. The insufficiency of hand-crafted features and priors to extract accurate semantic information leads to their unsatisfactory predictions in complex scenarios. The recent trend in SOD, deep learning-based models have delivered exceptional performance even in the presence of challenging issues such as multiple objects, scale variations, reflections and background clutter. The qualitative evaluation shows that even the most effective models demonstrated a lot of variations in performance on different varieties of challenging scenes. However, recent SOD models incorporating edge information, contextual information and/or combining discriminative saliency features perform better on quantitative measures. Moreover, multiple deep learning-based SOD approaches targeted at high processing efficiency are also discussed and compared with other representative SOD approaches. Several weakly-supervised models which discard the requirement of costly-to-construct pixel-accurate ground truth data for training the corresponding SOD models are also covered in detail. Easier and fast image labels, such as scribbles can be helpful for researchers to create larger-scale datasets with more focus on data selection issues rather than annotation-related issues. In the end, some future directions to enhance the current state-of-the-art in SOD are also discussed. 

## Figures and Tables

**Table 1:** Existing review publications on SOD from images and related research fields.

#	Title	Publ.	Focused Attentive Task	Coverage Span (upto)	Short Description
1	State-of-the-Art in Visual Attention Modeling [46]	TPAMI	Fixation prediction (FP)	2012	Reviewed traditional models for visual attention.
2	Salient Object Detection: A Benchmark [45]	TIP	FP and SOD	2014	Qualitatively evaluated selective heuristic FP and SOD models over seven datasets.
3	Attentive Systems: A Survey [47]	IJCV	General attention	2017	Application oriented review of attentive (SOD and FP) techniques.
4	Review of Visual Saliency Detection with Comprehensive Information [48]	TCSVT	RGB-D, Co-saliency and Video saliency	2018	Review of traditional, and learning-based models for all 3 SOD tasks.
5	Saliency prediction in the deep learning era: Successes and limitations [49]	TPAMI	FP	2018	Covered of FP models for still images and videos.
6	Salient Object Detection: A Survey [7]	CVM	SOD	2017	Reviewed early deep learning-based models for RGB images and heuristic models for 2-D, 3-D and 4-D images.
7	Salient Object Detection in the Deep Learning Era: An In-Depth Survey [44]	arXiv	SOD	2019	Compact coverage and attribute-based analysis of deep SOD models for RGB-images.
8	RGB-D Salient Object Detection: A Survey [50]	arXiv	RGB-D SOD	2020	Reviewed RGB-D based SOD and light field SOD models and benchmark their datasets.

**Table 2:** Comparison of salient object detection with other computer vision tasks (GT - Ground truth).

#	Task	Aim	GT Map	Vs. SOD
1	Fixation prediction	Finds where human look in a scene.	Several fixation dots in human fixation map.	Pixel-wise GT maps with clear boundaries are seldom used.
2	Image/Semantic segmentation	Assigns a label to each pixel in the image.	Each pixel has an associated category label.	Scope is the entire image, not just the salient objects.
3	Object proposals	Generates overlapping candidate region proposals.	Rectangular bounding-box annotation.	Objectness prior have been utilized in heuristic SOD models.
4	Object detection	To locate object(s) from fixed category list.	Rectangular bounding-box annotation.	Locates all instances of desired type, not just salient.
5	Salient object subitizing	Find existence and the number of salient objects.	Pixel accurate annotation with a count.	Indexing of individual objects as salient.

**Table 3:** The objective functions of different LR methods.

Model	Objective Function Terms				Short Descriptions
	Φ(.)	Ω(.)	Θ(.)	add’l. Constraint	
ULR model [124]	${∥L∥}_{*}$	${∥S∥}_{1}$	-	-	${∥.∥}_{*}$ is the nuclear norm and ${∥.∥}_{1}$ is the L1 regularizer.
SLR model [118]	${∥L∥}_{*}$	${∥S∥}_{1}$	-	$F=AH_{c}$	$A\in R^{D\times N}$ is a feature matrix and $H_{c}$ is a set of *N* prior values with one value each for a superpixel.
LSMD model [125]	${∥L∥}_{*}$	$\sum_{i}^{d}\sum_{j}^{n_{i}}v_{j}^{i}{∥S_{G_{j}^{i}}∥}_{2,\infty }$	–	–	Ω(.) represents a tree-structured sparsity regularizer. $v_{j}^{i}\geq 0$ weights the node $G_{j}^{i}$, $S_{G_{j}^{i}}\in R^{D\times |G_{j}^{i}|}$ where, *d* and $n_{i}$ represent # of tree levels and # of nodes per level, respectively.
SMD model [126]	${∥L∥}_{*}$	$\sum_{i}^{d}\sum_{j}^{n_{i}}v_{j}^{i}{∥S_{G_{j}^{i}}∥}_{p}$	$Tr({\mathrm{SM}}_{F}S^{T})$	–	Θ(.) represents a Laplacian sparsity regularizer. *p* is set to *∞*, $M_{F}$ is the Laplacian matrix.

**Table 5:** Popular deep architecture backbone networks for salient object detection.

Architecture	Year	Publication	Key Features	Layers	Representative Model
VGG [56]	2014	ICLR	Small size convolution kernels, More	13, 16, 19	[159,160]
			discriminative decision function.		
ResNet [145]	2016	CVPR	Much deeper network, residual	18, 34, 50, 101, 152	[161,162]
			modeling eases the training process		
			of a very deep network structure.		
DenseNet [163]	2017	CVPR	Less parameters, more reuse of features,	121,169, 201, 264	[164]
			better training relives from the vanishing		
			gradient and model degeneration problems.		
ResNext [165]	2017	CVPR	Homogeneous, multi-branch architecture,	101	[166]
			few hyper-parameter setting required.		

**Table 7:** Summary of Progressive feature refinement models.

Method	Publ.	Year	Backbone	Training Dataset	Strategy
Deep Hierarchical Saliency (DHSNet) [179]	CVPR	2016	VGGNet	MSRA10K [54] + DUT-OMRON [110]	Recurrent convolution based refinement.
Bi-directional Message Passing (BDMP) [159]	CVPR	2018	VGGNet	DUTS [174]	Enabled bi-directional message passing.
Progressive Attention Guided Recurrence (PAGR) [180]	CVPR	2018	VGGNet19	DUTS [174]	Spatial-/Channel-wise attention mechanism.
Pixel-wise Contextual Attention (PiCANet) [181]	CVPR	2018	VGGNet/ResNet50	DUTS [174]	Learned local and global pixel-wise contextual information.
Detect Globally Refine Locally (DGRL) [182]	CVPR	2018	ResNet50	DUTS [174]	Recurrence connections within ResNet50 blocks.
Reverse Attention (RAS) [160]	ECCV	2018	VGGNet	MSRA-B [54]	Residual connections with reverse attention mechanism.
Pooling-based Network (PoolNet) [183]	CVPR	2019	ResNet50	DUTS [174]	Pooling intensive approach.
Boundary-Aware Saliency (BASNet) [184]	CVPR	2019	ResNet-34	DUTS [174]	Saliency refinement based on residual network. Introduced novel losses.
Attention Feedback (AFNet) [177]	CVPR	2019	VGGNet16	DUTS [174]	Feedback mechanism in scale matching encoder-decoder pair.
Iterative Pathways Saliency (IPS) [185]	CVPR	2019	ResNet50	MSRA10K [54]	Iterative top-down/bottom-up inference.
Joint Deep features (JDF) [186]	ICCV	2019	VGG	MSRA-B [54]	Modelled interaction between side-features and predictions.
Gated Network (GateNet) [166]	arXiv	2020	ResNet/ResNeXt-101	DUTS [174]	Gates with encoder-decoder inputs to control message passing.
Spatial Attenuation Context (SACNet) [187]	ITCSVT	2020	ResNet-101	DUTS [174]	Used spatial attenuation context for SOD.
Multistage Interactive (MINet) [188]	CVPR	2020	VGG-16/ResNet-50	DUTS [174]	Mutual learning based interaction modules.

**Table 8:** Summary of Multi-task learning models.

Method	Publ.	Year	Backbone	Training Dataset	Strategy
Delving salient object subitizing (DSOS) [195]	ICCV	2017	VGGNet	SOS [69]	Salient object subitization assists SOD with the count of salient objects.
Revisiting Saliency Detection (RSDNet-R) [196]	CVPR	2018	ResNet101	PASCAL-S [158]	Gate based skip connections.
Boundary-Aware (BANet) [194]	ICCV	2019	ResNet50	DUTS [174]	Build three streams network to address selectivity-invariance delimma.
Stacked Cross refinement (SCRN) [162]	ICCV	2019	ResNet50	DUTS [174]	Let multiple tasks benefit each other.
Caption Saliency (CapSal) [147]	CVPR	2019	ResNet101	COCO-CapSal [147]/DUTS[174]	Utilized image captioning for SOD.
Saliency Semantic-segmentation (SSNet) [197]	ICCV	2019	Densenet169	PASCAL VOC 2012 [198]/DUTS [174]	Unified framework for weakly supervised semantic segmentation and SOD.
Edge Guided (EGNet) [199]	ICCV	2019	VGGNet/ResNet	DUTS [174]	Combined SOD features and Edge features at multiple scales.
Mutual Learning saliency (MLSL) [146]	CVPR	2019	VGGNet16	DUTS [174]	Mutual learning module based.
Pyramid Attention Edge (PAGE-Net) [44]	CVPR	2019	VGGNet16	MSRA10K [54]	Utilized Hierarchical attention mechanism.
Label Decomposition Framework (LDF) [148]	CVPR	2020	ResNet-50	DUTS [174]	Saliency mask decoupled for better edge and interior supervision.
$U^{2}$-Net [193]	PR	2020	RSU	DUTS [174]	Two-level nested U-structure which is trained from scratch.

**Table 9:** Summary of Other methods.

Method	Publ.	Year	Backbone	Training Dataset	Strategy
Recurrent FCN (RFCN) [201]	ECCV	2016	VGGNet	PASCAL VOC 2010 [198] + MSRA10K [54]	Utilized conventional saliency maps and recurrent FCN.
Recurrent Attention (RACDNN) [202]	CVPR	2016	VGGNet	DUT-OMRON [110] + NJU2000 [207] + RGBDSOD [208]	Spatial transform network to attend to image sub-regions.
Uncertain Conv Features (UCF) [203]	ICCV	2017	VGGNet	MSRA10K [54]	Introduced R-dropouts in encoder.
Deep Level-Sets (DLS) [204]	CVPR	2017	VGGNet	MSRA10K [54]	Level-set based loss function.
Stagewise Refinement Model (SRM) [205]	ICCV	2017	ResNet	DUTS [174]	Multi-stage refinement mechanism.
Multi-scale Refinement (MSRNet) [157]	CVPR	2017	ResNet	DUTS [174]	Multi-scale refinement mechanism.
High Resolution SOD (HRSOD) [206]	ICCV	2019	VGGNet	DUTS [174] + HRSOD [206]	High resolution SOD utilizing global cues for local refinement.

**Table 10:** Summary of Weakly-supervised/Pseudo-supervised.

Method	Publ.	Year	Backbone	Training Dataset	Supervision Source
Image Level Supervision (ILS) [174]	CVPR	2017	VGGNet	ImageNet [55]	Image-level tags.
Deep Unsupervised Saliency (DUS) [13]	CVPR	2018	ResNet101	MSRA-B [54]	Noisy saliency maps from four heuristic SOD.
Contour2Saliency C2S-Net [209]	ECCV	2018	VGGNet	MSRA10K [54]+Web	Contour information.
Weakly-Supervised Scribble Annotations (WSSA) [149]	CVPR	2020	VGGNet	Scribble-DUTS [149]	Scribble annotation based.

**Table 11:** Salient object detection datasets. Annotations (Annt): {BB = Bounding box, PW = pixel-wise object level, PWIL = pixel-wise instance level}. Property (Object): {ML= multiple, LG = large, SM = small, MD = moderate, CN = center, SA = similar in appearance among multiple images}, Property (Background(Bkg)): {CL = clean, SE = simple, CM = complex, RE = Repeat, TX = Texture images}, TR = training set and TE = testing set.

S.No	Dataset	Year	Publication	Images	Annt	Property		Resolution	
						Object	Bkg	Max(w,h)	Min(w,h)
1	MSRA-A [54]	2007	CVPR	20,840	BB	1-2, LG, CN	CL, SE	400	165
2	MSRA-B [54]	2007	CVPR	2500 (TR) + 2500 (TE)	BB	1-2, LG, CN	CL, SE	400	126
3	SED1 [218]	2007	CVPR	100	PW	1	SE	465	125
4	SED2 [218]	2007	CVPR	100	PW	2	CM	300	144
5	ASD [14]	2009	CVPR	1000	PW	1-2, MD, CN	CL, SE	400	165
6	SOD [217,219]	2010	CVPR-W	300	PW	ML, MD	CM	481	321
7	MSRA5K [54,95]	2011	CVPR	5000	PW	1	CL, SE	400	144
8	CSSD [103]	2013	CVPR	200	PW	1	CL, SE	400	139
9	ECSSD [103]	2013	CVPR	1000	PW	ML, LG	CL, SE	400	139
10	MSRA10K [54]	2013	CVPR	10,000	PW	1	CL, SE	400	144
11	DUT-OMRON [110]	2013	CVPR	5168	PW	ML, SM	CM	401	89
12	PASCAL-S [158]	2014	CVPR	850	PW	ML, MD	CM	500	151
13	HKU-IS [154]	2015	CVPR	3000 (TR) +1447 (TE)	PW	ML, MD	CM	401	100
14	DUTS [174]	2017	CVPR	10,553 (TR) + 5019 (TE)	PW	ML	CM	400	126
15	XPIE [220]	2017	CVPR	10,000	PWIL	1, MD	CM	500	130
16	ILSO [157]	2017	CVPR	1000	PWIL	ML, MD	CM	400	142
17	SOC [221]	2018	ECCV	6000	PWIL, OC	0-4+, MD	CM, TX	849	161
18	HRSOD [206]	2019	ICCV	1610 (TR) + 400 (TE)	PW	ML, MD	CM	10,240	600
19	DAVIS-S [206,222]	2019	ICCV	92	PW	ML, MD, SA	CM, RE	3840	720

**Table 12:** Quantitative Performance of recent state-of-the-art deep learning-based SOD methods on 5 popular datasets. Performance metrics of maximum F-measure, S-measure, E-measure, and Mean Absolute Error (MAE) is represented by $maxF_{\beta }$, $S_{m}$, $E_{m}$, and MAE, respectively. Superscript in the first column: “X”, “S”, “D” represent ResNeXt-101, ResNet-101 and DenseNet backbone. ↑ and ↓ indicate that the larger and smaller scores are better respectively.

Model	ECSSD [103]				HKU-IS [154]				DUT-OMRON [110]				DUTS-TE [174]				Pascal-S [158]			
	${\mathrm{maxF}}_{\beta }↑$	$S_{m}↑$	$E_{m}↑$	MAE ↓	${\mathrm{maxF}}_{\beta }↑$	$S_{m}↑$	$E_{m}↑$	MAE ↓	${\mathrm{maxF}}_{\beta }↑$	$S_{m}↑$	$E_{m}↑$	MAE ↓	${\mathrm{maxF}}_{\beta }↑$	$S_{m}↑$	$E_{m}↑$	MAE ↓	${\mathrm{maxF}}_{\beta }↑$	$S_{m}↑$	$E_{m}↑$	MAE ↓
VGG																				
ILS [174]	0.855	0.811	0.868	0.103	0.859	0.822	0.897	0.078	0.689	0.730	0.766	0.110	0.737	0.737	0.793	0.100	0.771	0.743	0.791	0.139
MSRNet [157]	0.911	0.895	0.918	0.054	0.914	0.903	0.940	0.040	0.782	0.808	0.827	0.073	0.829	0.839	0.848	0.061	0.858	0.841	0.854	0.081
NLDF [15]	0.905	0.875	0.912	0.063	0.902	0.878	0.929	0.048	0.753	0.817	0.770	0.080	0.812	0.816	0.855	0.065	0.833	0.804	0.842	0.099
Amulet [171]	0.915	0.894	0.912	0.059	0.899	0.886	0.915	0.050	0.743	0.781	0.784	0.098	0.778	0.804	0.803	0.085	0.841	0.821	0.831	0.098
UCF [203]	0.903	0.884	0.896	0.069	0.888	0.874	0.904	0.061	0.730	0.760	0.768	0.120	0.773	0.783	0.770	0.112	0.825	0.807	0.809	0.115
DSS [167]	0.899	0.873	0.907	0.068	0.916	0.878	0.935	0.040	0.781	0.790	0.844	0.063	0.825	0.824	0.885	0.056	0.843	0.795	0.848	0.096
PiCANet [181]	0.931	0.914	0.926	0.046	0.922	0.905	0.938	0.042	0.794	0.826	0.842	0.068	0.851	0.861	0.865	0.054	0.871	0.851	0.862	0.077
RAS [160]	0.921	0.893	0.922	0.056	0.913	0.887	0.931	0.045	0.787	0.814	0.849	0.062	0.831	0.839	0.864	0.059	0.838	0.795	0.837	0.104
C2S−Net [209]	0.910	0.893	0.914	0.054	0.895	0.882	0.927	0.048	0.757	0.7981	0.828	0.072	0.806	0.874	0.816	0.063	0.842	0.836	0.845	0.081
PAGR [180]	0.927	0.889	0.917	0.061	0.919	0.889	0.941	0.047	0.771	0.775	0.843	0.071	0.854	0.838	0.883	0.055	0.858	0.817	0.854	0.093
JDF [186]	0.927	0.906	0.931	0.049	0.920	0.903	0.943	0.039	0.801	0.821	0.862	0.057	0.832	0.825	0.860	0.058	0.856	0.841	0.853	0.082
BDMP [159]	0.929	0.910	0.915	0.044	0.927	0.906	0.938	0.039	0.792	0.809	0.839	0.064	0.854	0.850	0.862	0.048	0.854	0.845	0.845	0.073
CPD [173]	0.936	0.910	0.943	0.040	0.924	0.904	0.952	0.033	0.794	0.818	0.868	0.057	0.864	0.867	0.908	0.043	0.873	0.843	0.884	0.074
MLMS [146]	0.928	0.911	0.916	0.045	0.920	0.907	0.938	0.039	0.774	0.809	0.839	0.064	0.852	0.862	0.863	0.049	0.864	0.845	0.847	0.075
PAGE [44]	0.931	0.912	0.943	0.042	0.920	0.904	0.948	0.036	0.792	0.825	0.860	0.062	0.838	0.854	0.886	0.052	0.859	0.840	0.879	0.078
AFNet [177]	0.935	0.912	0.940	0.042	0.925	0.905	0.948	0.036	0.797	0.826	0.859	0.057	0.863	0.855	0.892	0.046	0.871	0.849	0.885	0.071
PoolNet−Edge[183]	0.941	0.917	0.942	0.041	0.931	0.911	0.951	0.033	**0.820**	0.832	0.863	**0.056**	**0.879**	0.866	0.894	0.041	0.868	0.851	0.873	0.071
EGNet [199]	0.942	0.918	0.941	0.041	0.926	0.911	0.940	0.035	0.808	**0.836**	**0.864**	**0.056**	0.877	**0.877**	0.894	0.044	0.870	0.847	0.872	0.077
MINet [161]	**0.943**	**0.919**	**0.947**	**0.036**	**0.932**	**0.914**	**0.955**	**0.030**	0.794	0.822	**0.864**	0.057	0.877	0.875	**0.912**	**0.039**	**0.882**	**0.855**	**0.898**	**0.065**
ResNet-50/ResNet-101/DenseNet/ResNeXt-101/RSU																				
SRM [205]	0.917	0.895	0.928	0.054	0.906	0.887	0.939	0.046	0.769	0.798	0.843	0.069	0.826	0.836	0.867	0.059	0.850	0.833	0.861	0.085
DGRL [182]	0.925	0.906	0.943	0.043	0.914	0.896	0.947	0.038	0.779	0.810	0.850	0.063	0.828	0.842	0.899	0.050	0.860	0.839	0.881	0.075
BASNet [184]	0.942	0.916	0.921	0.037	0.93	0.908	0.947	0.033	0.805	0.836	0.869	0.056	0.859	0.866	0.884	0.048	0.863	0.837	0.853	0.077
$CapSal^{S}$ [147]	0.862	0.826	0.866	0.074	0.884	0.850	0.907	0.058	0.639	0.674	0.703	0.096	0.823	0.815	0.866	0.062	0.869	0.837	0.878	0.074
PoolNet−Edge [183]	0.949	0.926	0.948	0.035	0.936	0.918	0.958	0.029	0.830	0.831	0.873	0.053	0.893	0.874	0.909	0.036	0.884	0.864	0.887	0.064
BANet [194]	0.945	0.924	0.953	0.035	0.930	0.913	0.955	0.032	0.803	0.832	0.865	0.059	0.872	0.879	0.907	0.040	0.879	0.853	0.889	0.070
SCRN [162]	0.950	0.927	0.942	0.037	0.935	0.917	0.954	0.033	0.811	0.837	0.869	0.056	0.888	0.885	0.901	0.040	0.890	0.867	0.888	0.065
$DSRNet^{D}$ [164]	0.950	0.922	0.953	0.031	0.939	0.915	0.954	0.027	0.822	0.829	0.933	0.053	0.891	0.863	0.918	0.036	0.888	0.798	0.85	0.068
LDF [148]	0.950	0.923	0.950	0.034	0.940	0.920	0.961	0.027	0.821	0.839	0.881	0.051	0.896	0.879	0.923	0.034	0.875	0.862	0.904	**0.059**
$U^{2}-Net^{RSU}$ [193]	0.951	0.928	0.925	0.032	0.934	0.913	0.945	0.031	0.822	0.846	0.871	0.054	0.872	0.860	0.883	0.045	0.861	0.844	0.850	0.074
MINet [161]	0.947	0.925	0.953	0.033	0.935	0.920	0.961	0.028	0.810	0.833	0.873	0.055	0.884	0.884	0.917	0.037	0.882	0.857	0.899	0.064
$GateNet^{X}$ [166]	0.952	0.929	-	0.035	0.943	**0.925**	-	0.029	0.829	**0.848**	-	0.051	**0.898**	**0.895**	-	0.035	**0.888**	**0.865**	-	0.065
$SACNet^{S}$ [187]	**0.954**	**0.930**	**0.958**	**0.028**	**0.945**	**0.925**	**0.969**	**0.023**	**0.832**	0.846	**0.883**	**0.050**	**0.898**	0.878	**0.920**	**0.032**	0.876	0.801	**0.902**	0.070

**Table 13:** Average running time of several salient object detection (SOD) models.

Models	SF [106]	MR [110]	RBD [117]	DRFI [92]	MCDL [150]	AMULet [171]	UCF [203]	C2S-Net [209]
Time(s)	0.16	0.25	0.25	9	2.41	0.07	0.046	0.034
GPU Support	No	No	No	No	Yes	Yes	Yes	Yes
Learning	No	No	No	CML	DL	DL	DL	DL
Code	C++	Matlab	Matlab	Matlab	Caffe	Caffe	caffe	caffe
**Models**	**PiCANet [181]**	**RAS [160]**	**PoolNet [183]**	**AFNet [177]**	**EGNet [199]**	**SCRN [162]**	**CPD [173]**	**BASNet [184]**
Time(s)	0.19	0.0291	0.033	0.023	0.11	0.032	0.016	0.014
GPU Support	Yes	Yes	Yes	Yes	Yes	Yes	Yes	Yes
Learning	DL	DL	DL	DL	DL	DL	DL	DL
Code	caffe	caffe	caffe	pytorch	pytorch	pytorch	prtorch	pytorch
